# Comparative transcriptome analysis of differentially expressed genes of *Medicago falcata* L. breeding lines response to saline-alkaline stress

**DOI:** 10.1186/s12870-025-06599-3

**Published:** 2025-05-13

**Authors:** Hua Chai, Xiaolong Wang, Zhao Yang, Shasha Li, Yanxia Xu, Yue Wu, ZhongBao Shen

**Affiliations:** 1 Branch of Animal Husbandry and Veterinary of Heilongjiang Academy of Agricultural Sciences, Qiqihar, 161005 China; 2 Heilongjiang Academy of Agricultural Sciences Postdoctoral Programme, Harbin, 150086 China; 3 Institute of Forage and Grassland Sciences, Heilongjiang Academy of Agricultural Sciences, Harbin, 150086 China

**Keywords:** *Medicago falcata* L., Salt-alkali stress, RNA-seq, Transcription factor, WGCNA

## Abstract

**Background:**

Salt-alkali stress is an abiotic stress that inhibits crop growth and reduces yield. It significantly affects various physiological processes in plants, including photosynthesis, osmotic regulation, and antioxidant defense. However, studies on the transcriptional response mechanisms of *Medicago falcata* L. under salt-alkali stress are limited. In this study, RNA-seq technology was used to analyze differentially expressed genes (DEGs) in salt-alkali tolerant *M.falcata* breeding lines (LM18) and the salt-alkali sensitive Hulunbeier (HL) under salt-alkali stress. Furthermore, physiological indicators such as chlorophyll content, proline accumulation, and superoxide dismutase (SOD) activity were assessed to compare the responses of LM18 and HL to salt-alkali stress. By integrating transcriptomic and physiological analyses, this study provides new insights into the physiological and molecular regulatory mechanisms of *M. falcata* in response to salt-alkali stress.

**Results:**

The results showed that compared to the untreated controls, 10,289 and 2,478 DEGs were detected in *LM18* and *HL M.falcata* seedlings, with 788 shared DEGs detected in both. GO functional analysis classified these DEGs into three categories: Biological Process, Cellular Components, and Molecular Functions, with significant enrichment in GO terms such as “response to osmotic stress”, “intramolecular oxidoreductase activity” and “antioxidant activity”. Kyoto Encyclopedia of Genes and Genomes (KEGG) pathway analysis revealed the involvement of these DEGs in key metabolic pathways, including “Phenylpropanoid biosynthesis”, “Plant hormone signal transduction”, “Plant-pathogen interaction”, “Isoflavonoid biosynthesis”, “Circadian rhythm-plant” and “Photosynthesis—antenna proteins”. Physiological indicators and membership function analysis confirmed that LM18 has greater salt-alkali tolerance than HL. Transcription factor analysis identified 42 transcription factor families, with the ERF family being the most abundant, followed by MYB-related, WRKY, bHLH, and MYB families. Weighted Gene Co-expression Network Analysis (WGCNA) showed that the MEturquoise module exhibited a significant positive correlation with salt-alkali stress and several physiological indicators. Module gene network analysis and GO enrichment revealed that *MS.gene64536*(MYBP), *MS.gene76249*(SRM1) and *MS.gene049843* (MPK3) have functions related to “response to salt stress” and “positive regulation of response to salt stress”, suggesting their key roles in salt-alkali tolerance in *M.falcata*. All three genes were upregulated in the salt-alkali tolerant LM18.

**Conclusions:**

The GO terms and KEGG pathways significantly enriched in *LM18* involved a significantly higher number of DEGs compared to *HL*, suggesting a more robust and effective mechanism in LM18. These findings highlight the robust molecular and physiological adaptations of LM18 in response to salt-alkali stress.

**Supplementary Information:**

The online version contains supplementary material available at 10.1186/s12870-025-06599-3.

## Introduction

Soil salinization is a significant environmental issue that constrains agricultural and pastoral development. Globally, saline-alkaline land covers approximately one billion square kilometers, and this area continues to expand, causing extensive environmental damage and severely reducing crop yields [1]. Salt-alkali stress is one of the abiotic stress factors affecting plant growth and development, inducing ionic toxicity, osmotic stress, oxidative stress, and increased rhizosphere pH, which leads to high pH stress. The combined effects of these stresses disrupt numerous physiological and metabolic processes, inhibit plant growth, and can ultimately lead to premature senescence or plant death [2, 3]. Developing salt-alkali tolerant crops remains an effective strategy to mitigate soil salinization. In-depth investigation and utilization of salt-alkali tolerance genes can provide molecular insights into the underlying mechanisms of salt-alkali resistance. However, challenges such as the limited availability of salt-alkali tolerant genes and germplasm resources persist. Identifying key genes involved in the salt-alkali stress response and elucidating the molecular mechanisms underlying plant tolerance is critical for developing salt-alkali tolerant crops, provides a potential strategy to mitigate the impact of saline-alkaline soils on agriculture [4].

The saline-alkaline components in soil primarily include neutral salts such as NaCl and Na₂SO₄, which induce salt stress, and alkaline salts such as NaHCO₃ and Na₂CO₃, which cause alkaline stress [5, 6]. Excessive Na⁺ from neutral salts disrupts plant metabolic processes [4] and reduces soil water potential and osmotic potential [7, 8]. Significant progress has been made in understanding the molecular mechanisms underlying plant responses to NaCl stress [9, 10]. Studies have demonstrated that plants adapt to salt stress caused by NaCl by accumulating osmolytes such as soluble sugars and proline, thereby regulating cellular osmotic pressure [11, 12]. In contrast, alkaline salts induce high pH damage through CO₃^2^⁻ and HCO₃⁻ [13, 14], triggering the synthesis of reactive oxygen species (ROS) and malondialdehyde, which disrupt plant cell membranes and intracellular components [15, 16]. Consequently, alkaline stress causes more severe morphological and physiological damage in plants compared to neutral salts [17, 18]. Alkaline salts also exert a greater inhibitory effect on photosynthesis, leading to intensified oxidative damage [19–22]. Despite these impacts, the molecular mechanisms underlying plant responses to alkaline stress remain underexplored. Recent studies in *Sorghum bicolor* have identified a key negative regulator, AT1, which modulates salt-alkali tolerance by altering H₂O₂ distribution between plant cells through the phosphorylation of aquaporins [23, 24]. Studies on the response of *Medicago sativa* L. to salt-alkali stress have revealed significant enrichment of amino acid biosynthesis, flavonoid biosynthesis, and MAPK signaling pathways in salt-alkali tolerant varieties [25].

In recent years, advancements in transcriptome sequencing technology have significantly enhanced the understanding of plant responses to various abiotic stresses [26–28]. RNA-Seq high-throughput sequencing directly sequences cDNA derived from mRNA, enabling the detection of global transcriptional activity at single-nucleotide resolution. This technique rapidly and comprehensively identifies almost all transcripts under specific conditions, making it a powerful tool for uncovering gene expression related to plant stress responses. RNA-Seq has been widely applied to plants such as *Medicago sativa*, *Leymus chinensis*, and *Arabidopsis thaliana*. Pang et al. [29] conducted RNA sequencing and WGCNA analysis on the salt-tolerant cotton variety Lu Mian 28 and the salt-sensitive variety Zhong Mian Suo 12 under salt stress. They identified 114 transcription factors (TFs) and 11 key genes related to salt tolerance. Gan et al. [30] performed transcriptomic analysis on tomato roots during the early stages of salt stress and identified 10,588 differentially expressed genes (DEGs), including genes involved in hormone signaling, amino acid metabolism, and cell cycle regulation. Ren et al. [31] performed RNA sequencing on salt-alkali mixed stress-treated salt-tolerant soybean Heinong 531 and salt-sensitive soybean 20_1846, identifying 200 DEGs related to salt-alkali tolerance, of which 29 key DEGs were involved in 14 pathways responding to salt-alkali stress. Ni et al. [32] analyzed transcriptome data of *Hibiscus hamabo* under salt treatment and identified the key transcription factor HhWRKY79. Overexpression of HhWRKY79 in Arabidopsis was shown to enhance salt tolerance in transgenic plants. Ji et al. [33] performed RNA sequencing on banana root tissues under salt stress and found that multiple MYB and HAK TFs were upregulated, while the expression of most AP2/ERF and WRKY TFs was downregulated. An et al. [34] conducted transcriptome sequencing of Zhaodong alfalfa under salt-alkali stress and identified DEGs, elucidating the molecular mechanisms underlying its salt-alkali tolerance. Similarly, Cao et al. [35] used Illumina HiSeq 2500 high-throughput sequencing to identify miRNAs in *Medicago truncatula* under salt-alkali stress, providing valuable insights into the molecular mechanisms of salt-alkali tolerance and shedding light on the proposed roles of miRNAs in plant stress resistance.

*Medicago falcata* L. is a perennial leguminous forage crop with strong adaptability, excellent salt-alkali tolerance, and high forage quality, making it valuable for feeding, ecological, and genetic breeding purposes [36]. With the increasing severity of soil salinization, *M. falcata*, which has long been exposed to saline-alkali environments, has gradually developed salt-alkali tolerance through evolutionary adaptation to its habitat. Studies have shown that *M. falcata* contains numerous germplasm resources with robust salt tolerance, offering significant potential for alfalfa salt tolerance breeding. However, research on the salt-alkali tolerance of *M. falcata* has primarily focused on neutral salts (NaCl and Na₂SO₄), with less emphasis on alkaline salts (Na₂CO₃ and NaHCO₃). Since the genomic information of *M.falcata* has not yet been published, research on its salt-alkali tolerance rely largely on genomic data from model leguminous plants such as *Medicago truncatula* and *Medicago sativa*. Currently, research on the salt-alkali tolerance of *M. falcata* remains primarily at the morphological and physiological levels. It is imperative to investigate the response mechanisms of *M.falcata* to salt-alkali stress and to identify highly expressed functional genes and novel regulatory pathways involved in this process. Such exploration will provide new perspectives and insights into the molecular basis of salt-alkali tolerance in *M.falcata*. Based on the findings of this study, new strategies for the agricultural development of saline-alkaline lands can be proposed—for example, enhancing the expression of specific genes to improve the salt-alkali tolerance of *M.falcata*. This would offer both theoretical and technical support for the efficient utilization of saline-alkaline soils. The salt-alkali-tolerant genotype LM18 used in this study is likely to harbor genes associated with salt-alkali tolerance, thereby offering valuable genetic resources for the identification of candidate tolerance genes. These findings can further support modern breeding approaches, such as marker-assisted selection (MAS). Meanwhile, comparative analysis with the salt-alkali-sensitive genotype HL will enable the elucidation of genetic differences under stress conditions, thus contributing to a better understanding of the genetic basis underlying salt-alkali tolerance in *M.falcata*. By comparing physiological and molecular responses between salt-alkali tolerant LM18 and salt-alkali sensitive HL under salt-alkali stress, the key genes and regulatory pathways conferring tolerance can be uncovered. In this study, two *M.falcata* varieties, salt-alkali tolerant LM18 and salt-alkali sensitive HL, previously identified through screening, were used as experimental materials. Plants were subjected to 150 mM NaHCO₃ and Na₂CO₃ treatments for 0 (CK), 1, 6, and 12 h. Transcriptome sequencing was performed using the NovaSeq X Plus platform to compare gene expression differences between the two varieties under salt-alkali stress and control conditions. Analysis of salt-alkali responsive genes identified candidate genes associated with salt-alkali tolerance. These results provide a foundation for enhancing the adaptability of *M.falcata* to saline-alkaline environments and offer valuable insights into the molecular mechanisms governing salt-alkali tolerance.

## Materials and methods

### Plant growth and treatment

The experimental materials used in this study were *Medicago falcata* L. cv. Longmu No.18 (LM18) and Hulunbeier (HL), provided by the Branch of Animal Husbandry and Veterinary of Heilongjiang Academy of Agricultural Sciences. Hereafter, LM18 and HL refer to the salt-alkali tolerant breeding lines and the salt-alkali sensitive control sample, respectively. Healthy and viable *M.falcata* seeds were selected, disinfected with 70% ethanol for 30 s, and further sterilized with 5% NaClO for 10 min. The seeds were then rinsed 4–5 times with distilled water. The cleaned seeds were evenly placed on glass petri dishes lined with moistened filter paper and incubated in a controlled climate chamber at 25 °C/15 °C (day/night) with 70% humidity in darkness to induce germination. After cotyledon emergence, the seedlings were transferred to alfalfa cultivation troughs and irrigated daily with a modified Hoagland nutrient solution to ensure healthy growth. When the seedlings reached the 4–5 leaf stage, salt-alkali stress treatment was applied. The salt-alkali solution consisted of 150 mM NaHCO₃ and Na₂CO₃ mixed at a 9:1 ratio. This specific ratio was determined based on previous studies, as it establishes a defined pH and ionic environment that not only reflects a certain level of salt-alkali stress intensity but also maintains relative ionic stability in the solution. This approach enables a more precise assessment of plant tolerance thresholds and physiological responses under the combined effects of salt and alkali stress. Seedlings of the LM18 and HL of *M.falcata* were subjected to salt-alkali stress treatment using a mixed alkaline solution composed of 150 mM NaHCO_3_ and Na_2_CO_3_ at a molar ratio of 9:1. Leaf samples were collected at four time points: 0, 1, 6, and 12 h after treatment. For each time point, three biological replicates were collected, resulting in a total of 24 samples. Leaf tissues were rapidly harvested, immediately frozen in liquid nitrogen, and stored at -80 °C for subsequent physiological measurements and RNA extraction. These samples were used for both physiological assays and transcriptomic analyses to investigate the molecular and physiological responses of *M.falcata* under salt-alkali stress.

### RNA extraction, library preparation, and sequencing

#### RNA extraction

To ensure the reliability and scientific rigor of the experimental results, three biological replicates were established for each treatment and for both experimental genotypes. A total of 24 samples were collected for RNA extraction, comprising four treatments (150 mM NaHCO_3_ and Na_2_CO_3_ for 0, 1, 6 and 12 h). RNA extraction was performed using TRIzol Reagent following the manufacturer's instructions (Invitrogen). Subsequently, the extracted RNA underwent quality assessment using the 5300 Bioanalyzer (Agilent Technologies) and was quantified with the ND-2000 (NanoDrop Technologies). Only high-quality RNA samples meeting the criteria of OD260/280 = 1.8–2.2, OD260/230 ≥ 2.0, RIN ≥ 8.0, 28S:18S ≥ 1.0, and > 1 μg were utilized for constructing the sequencing library.

#### Library preparation and sequencing

RNA purification, reverse transcription, library construction, and sequencing were conducted at Shanghai Majorbio Biopharm Biotechnology Co., Ltd. in Shanghai, China, following the guidelines of Illumina (San Diego, CA). The RNA-seq transcriptome libraries for LM18 and HL were constructed using 1 μg of total RNA with the Illumina® Stranded mRNA Prep Ligation Kit. Initially, messenger RNA was isolated via polyA selection with oligo (dT) beads, fragmented using a fragmentation buffer, and then subjected to double-stranded cDNA synthesis employing a SuperScript double-stranded cDNA synthesis kit (Invitrogen, CA) with random hexamer primers from Illumina. The cDNA was then subjected to end-repair, phosphorylation, and 'A' base addition following Illumina's standard library construction protocol. Target fragments of approximately 300 bp were size-selected using a 2% Low Range Ultra Agarose gel, and the libraries were amplified by PCR for 15 cycles with Phusion DNA polymerase. Library quantification was performed using Qubit 4.0, and paired-end sequencing (2 × 150 bp) was conducted on the NovaSeq X Plus sequencer. The raw paired-end reads were trimmed and quality-filtered using fastp [37] with default parameters. High-quality reads were aligned to the reference genome in orientation mode using HISAT2 [38]. Subsequently, the mapped reads of each sample were assembled via StringTie [39] in a reference-based approach. Subsequently, the quality-controlled sequencing data were evaluated by calculating the Q30 value and GC content.

### Analysis of DEGs and functional enrichment

For the identification of DEGs between two distinct varieties, the expression level of each transcript was determined using the transcripts per million reads (TPM) methodology. In this study, DEGs were defined as genes that exhibited significant expression changes in response to salt-alkali stress within each genotype (LM18 or HL), thereby contributing to the plants’ adaptive responses to the adverse alkaline environment. Gene abundances were quantified utilizing RSEM [40]. Subsequently, DESeq2 [41] was employed for the analysis of differential expression. DEGs meeting the criteria of |log2FC|≥ 1 and FDR < 0.05 (DESeq2) or FDR < 0.001 (DEGseq) were deemed as significant DEGs. Furthermore, functional enrichment analyses involving Gene Ontology (GO) and Kyoto Encyclopedia of Genes and Genomes (KEGG) pathways were conducted to identify DEGs significantly enriched in GO terms and metabolic pathways at a Bonferroni-corrected *P*-value < 0.05 compared to the whole-transcriptome background. GO functional enrichment analysis was performed using Goatools, while KEGG pathway analysis was conducted with Python scipy software. GO database is a widely used tool for the functional annotation and classification of genes and gene products. It categorizes gene functions into three main aspects: molecular function (MF), cellular component (CC), and biological process (BP). GO enrichment analysis enables the identification of biological processes involved in the response to salt-alkali stress, the subcellular localization of gene products, and their associated molecular functions. The KEGG database is another important resource that facilitates the analysis of metabolic and signaling pathways involving gene products. KEGG pathway enrichment analysis helps elucidate the biochemical processes occurring in response to salt-alkali stress, including which metabolic pathways are activated or suppressed, and which signaling pathways are involved in stress perception and response mechanisms.

### Measurement of physiological indicators

A total of 24 samples were collected for measurement of physiological indicators, comprising four treatments (150 mM NaHCO_3_ and Na_2_CO_3_ for 0, 1, 6 and 12 h), two varieties of leaves, and three biological replications. Chlorophyll is an essential component of photosynthesis and serves as a critical indicator for evaluating plant growth status and photosynthetic efficiency. Under salt-alkali stress, chlorophyll plays a key role in the light reaction of photosynthesis, converting absorbed light energy into chemical energy while mitigating photodamage caused by excessive light energy. Osmoregulatory substances are crucial for maintaining intracellular osmotic balance and ensuring normal cellular function under abiotic stress. For instance, proline accumulation under salt-alkali stress contributes to osmotic homeostasis, allowing plants to sustain water absorption and cell turgor pressure, thereby supporting normal growth and development in saline-alkali environments. ROS are continuously generated as byproducts of normal cellular metabolism. However, under salt-alkali stress, excessive ROS accumulation can lead to oxidative damage in plant cells. The plant antioxidant defense system, comprising enzymatic antioxidants such as superoxide dismutase (SOD), peroxidase (POD), and catalase (CAT), plays a vital role in scavenging excess ROS and protecting cells from oxidative stress. These antioxidant enzymes catalyze specific biochemical reactions that eliminate ROS generated under stress conditions, such as superoxide anions (O_2_^−^) and hydrogen peroxide (H_2_O_2_). By mitigating oxidative stress, these enzymes help maintain cellular redox homeostasis, preserve membrane integrity, and ensure the normal physiological functions of plant cells under adverse environmental conditions. All physiological measurements were conducted following the principles and techniques outlined in “Principles and Techniques of Plant Physiological and Biochemical Experiments” [42]. Chlorophyll content was measured using the extraction-colorimetric method, MDA content was quantified via the modified thiobarbituric acid (TBA) method, proline content was determined by the ninhydrin method, peroxidase (POD) activity was assessed using the guaiacol method, SOD activity was evaluated by the nitroblue tetrazolium (NBT) method, and CAT activity was measured by the ammonium molybdate colorimetric method, GSH content by the colorimetry method, Soluble Protein content by Coomassie brilliant blue colorimetry, Soluble Sugar content by the anthrone method, and H_2_O_2_ content by spectrophotometry method.

### Analysis methods of physiological indicators data

The data were expressed as mean ± standard deviation and variance analysis was performed to identify significant differences. At a significance level defined as α = 0.05, Duncan's test was used to determine the least significant difference (LSD) of the means. The experimental data were analyzed using SPSS software for variance analysis, and principal component analysis (PCA) was conducted using standardized values. Finally, the membership function method was employed to evaluate the salt-alkali tolerance of the two varieties. The membership function value for each variety was calculated using the formula:

$$U(Xi) = (Xi - Xmin) / (Xmax - Xmin), where i = 1, 2, 3, ..., n.$$

In this formula, *U*(*X*_*i*_) represents the membership function value of the *i*-th indicator for the tested variety; *X*_i_ represents the measured value of the *i*-th indicator; *X*_*min*_ and *X*_*max*_ represent the minimum and maximum values of the i-th indicator across all tested varieties.

### Weighted gene co-expression network analysis (WGCNA)

WGCNA is a powerful bioinformatics method used to analyze gene expression data by constructing co-expression networks. In the context of salt-alkali stress research, WGCNA facilitates the identification of gene modules and hub genes that are significantly correlated with salt-alkali tolerance traits. This approach provides valuable insights into the complex regulatory networks and molecular mechanisms underlying plant responses to salt-alkali stress. WGCNA was implemented utilizing the WGCNA package in R [43]. A co-expression network was constructed from all DEGs using the WGCNA package in R [43]. Modules were harvested utilizing the automatic network construction function, with modules grouped into blocks based on the default settings. The eigengene values for each module were calculated and used to assess their correlation with physiological indicators. Additionally, total connectivity, intramodular connectivity, kME (modular membership), and *p*-values of the DEGs were calculated.

### Identification of putative Transcription Factors (TFs)

All genes were searched against the Plant Transcription Factor Database (plant TFDB version 4.0, http://planttfdb.cbi.pku.edu.cn) using BLAST to identify putative TFs. TF information was annotated based on the comparative results.

### qRT-PCR analysis

Total RNA from the samples was used as a template for reverse transcription using the HiScript Q RT SuperMix for qPCR (+ gDNA wiper) kit (Vazyme, China). Nine gene-specific primers were designed using the tool on the NCBI website (https://www.ncbi.nlm.nih.gov/tools/primer-blast/), with β-actin gene expression serving as the normalizer. In the qRT-PCR experiment, the primer sequences used are crucial for ensuring the accuracy of the experimental results. The specific primer sequences are listed in Table S1 (Additional file 1). qRT-PCR was done on an ABI7300 Real-Time PCR System (Applied Biosystems, USA) utilizing ChamQ SYBR Color qPCR Master Mix (2X) (Vazyme, China). Relative gene expression levels were evaluated utilizing the 2^−ΔΔCT^ method.

## Results

### Identification of DEGs under salt-alkali stress

RNA-Seq technology was used to perform transcriptome sequencing of LM18 and HL under salt-alkali treatments for 0 (Control Check, CK), 1, 6, and 12 h, yielding 183.6 Gb of clean data, with a Q30 base percentage exceeding 93.3% and a GC content of approximately 42% (Additional file 2: Table S2). Correlation analyses of LM18 (Additional file 3: Figure S1) and HL (Additional file 4: Figure S2) indicated good consistency among the replicate data at the same treatment time point.

**Fig. 1:**
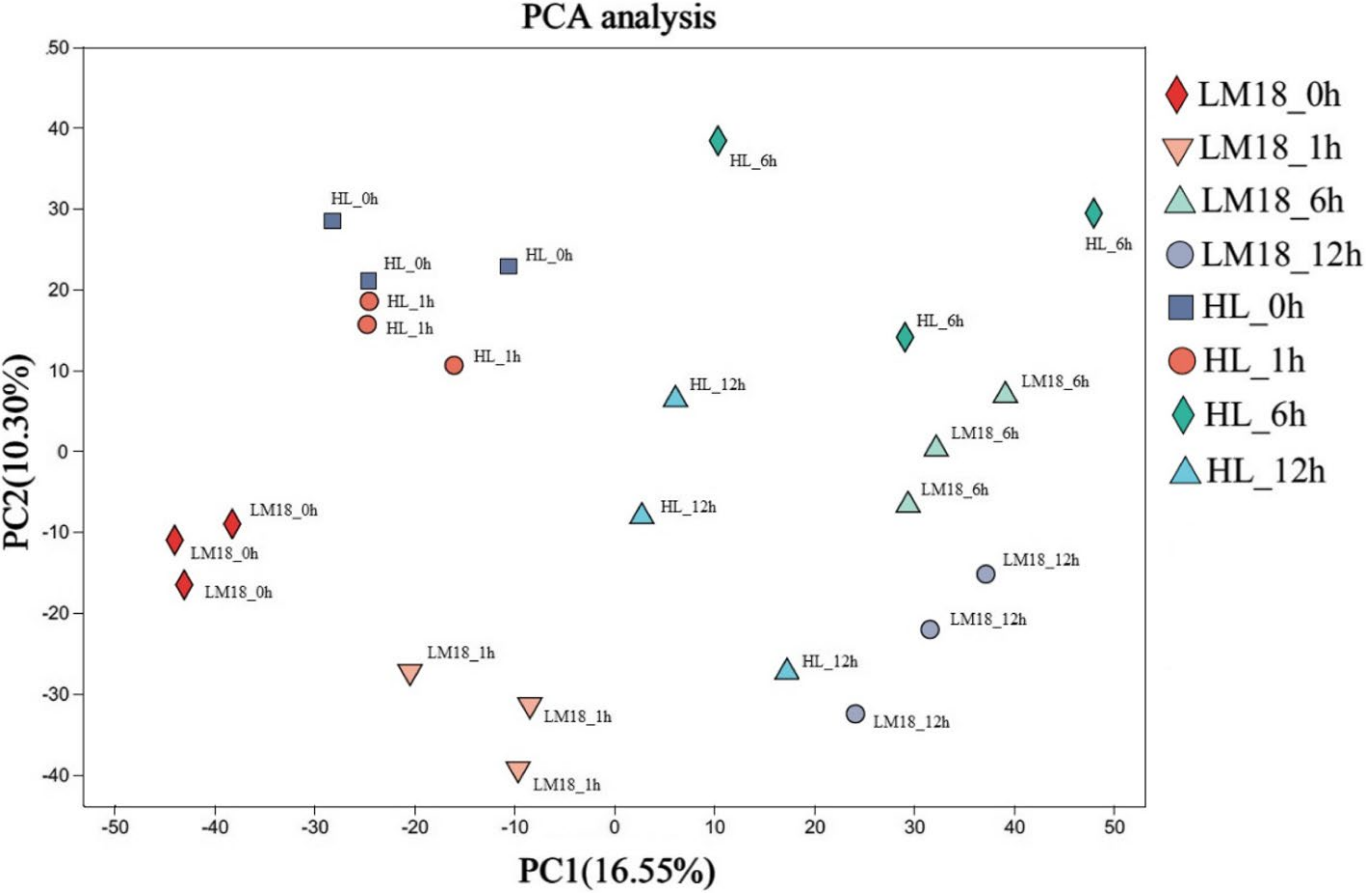
Principal component analysis of LM18 and HL

**Fig. 2:**
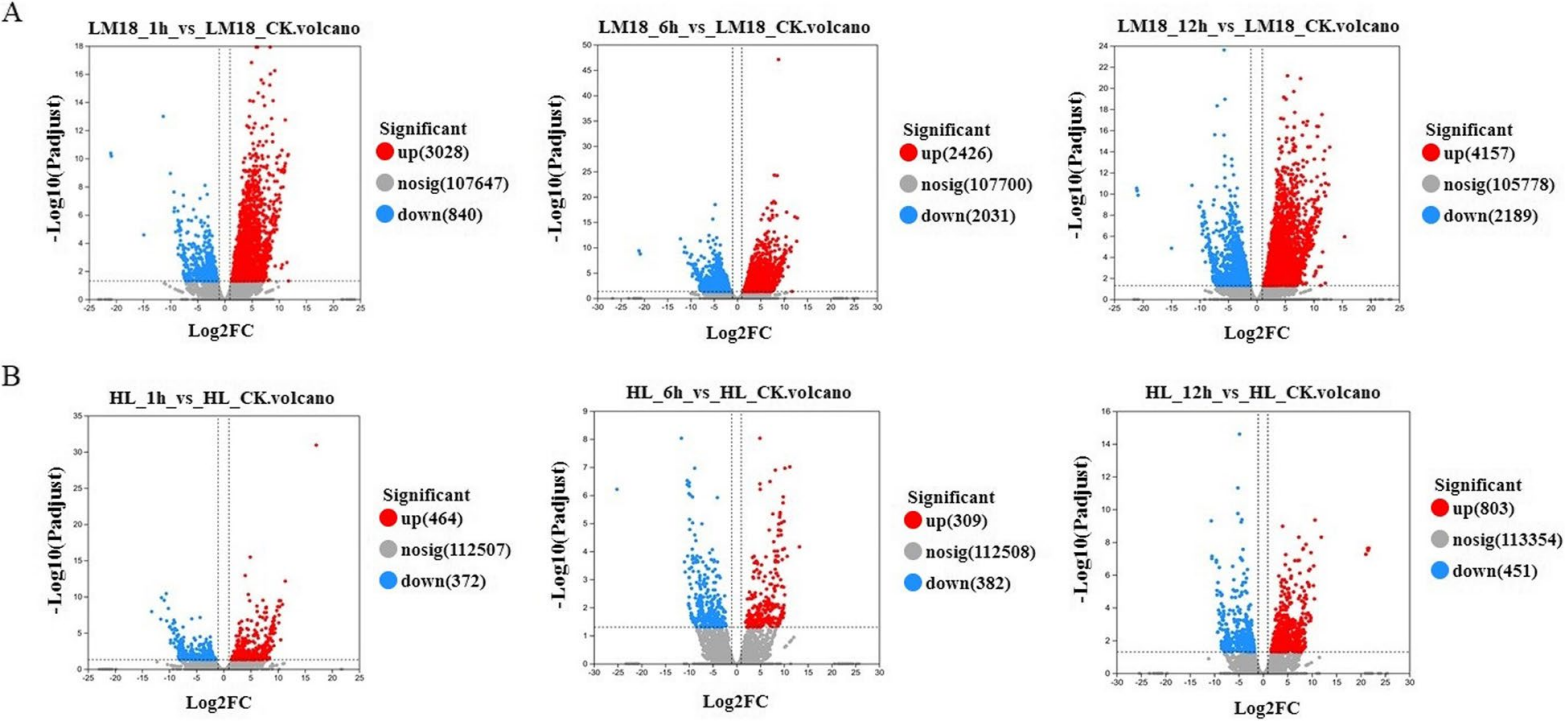
Volcano plots of differentially expressed genes in LM18 and HL. (**A**: The volcano maps of differentially expressed genes of LM18 at 1 h, 6 h and 12 h were obtained from CK(0 h), respectively. **B**: The volcano maps of differentially expressed genes of HL at 1 h, 6 h and 12 h were obtained from CK(0 h), respectively. Red points represent up-regulated genes, Blue points represent down-regulated genes, Grey points represent non-significantly genes.)

**Fig. 3:**
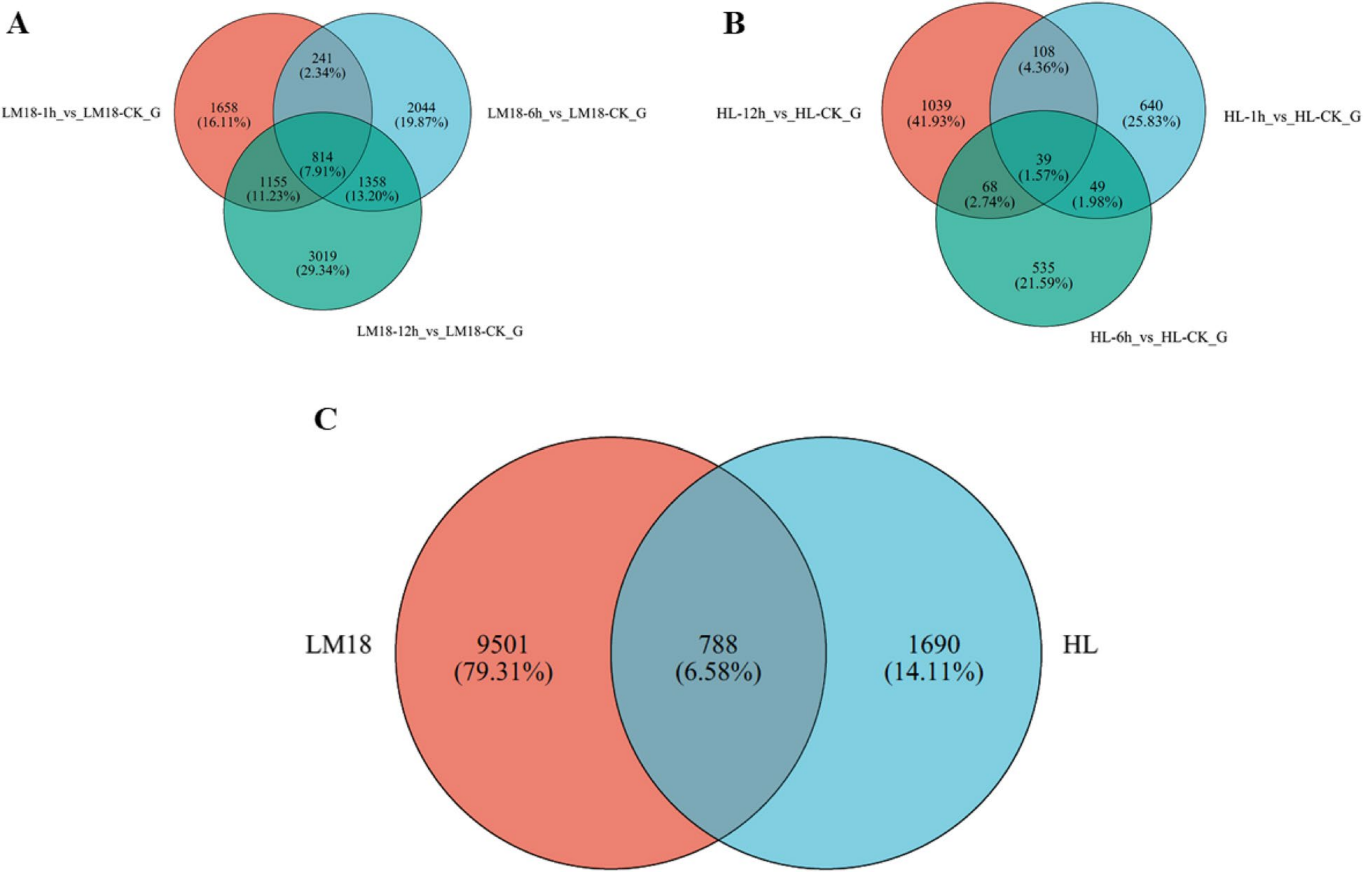
Venn diagrams of differentially expressed genes in LM18 and HL (**A**: Venn diagram of DEGs in LM18; **B**: Venn diagram of DEGs in HL; **C**: Venn diagram of DEGs in both LM18 and HL)

To examine the time-dependent transcriptional changes in LM18 and HL after salt-alkali treatment, we conducted PCA and observed significant differences in PC values between LM18 and HL. The expression change trends at the time points after salt-alkali treatment were similar for LM18 and HL, with only slight changes at 1 h and more pronounced changes at 6 h (Fig. 1). DEGs were identified using the criteria of FDR < 0.01 and |log2FC|≥ 1 (*p* < 0.05). In the LM18 transcriptome, a total of 10,289 DEGs were identified. Compared to the 0 h, 3,028, 2,426, and 4,157 genes were upregulated, and 840, 2,031, and 2,189 genes were downregulated at 1, 6, and 12 h, respectively (Fig. 2A), with 814 DEGs commonly expressed across the time points (Fig. 3A). In the HL transcriptome, 2,478 DEGs were identified, with 464, 309, and 803 genes upregulated and 372, 382, and 451 genes downregulated at 1, 6, and 12 h, respectively, compared to the 0 h (Fig. 2B), with 39 DEGs commonly expressed across the time points (Fig. 3B). The top 10 DEGs that were significantly upregulated under salt-alkali stress in LM18 and HL, respectively, compared to their corresponding controls at 0 h, are listed in Table S3 (Additional file 5). Under salt-alkali stress, the absolute number of DEGs generated by LM18 was 315.21% higher than that of HL. A comparative analysis of DEGs in response to salt-alkali stress between LM18 and HL (Fig. 3C) revealed 11,979 DEGs, with 788 DEGs detected in both LM18 and HL. These genes are preliminarily hypothesized to constitute the common salt-alkali response mechanism in *M.falcata* with varying salt-alkali tolerance, suggesting that they may not be the primary genes responsible for the differences in salt-alkali tolerance between the varieties. LM18 possessed 9,501 unique DEGs, significantly more than HL (1,690), which may be key gene groups determining the differences in salt-alkali tolerance between LM18 and HL.

### Functional analysis of DEGs in LM18 and HL under salt-alkali stress

**Fig. 4:**
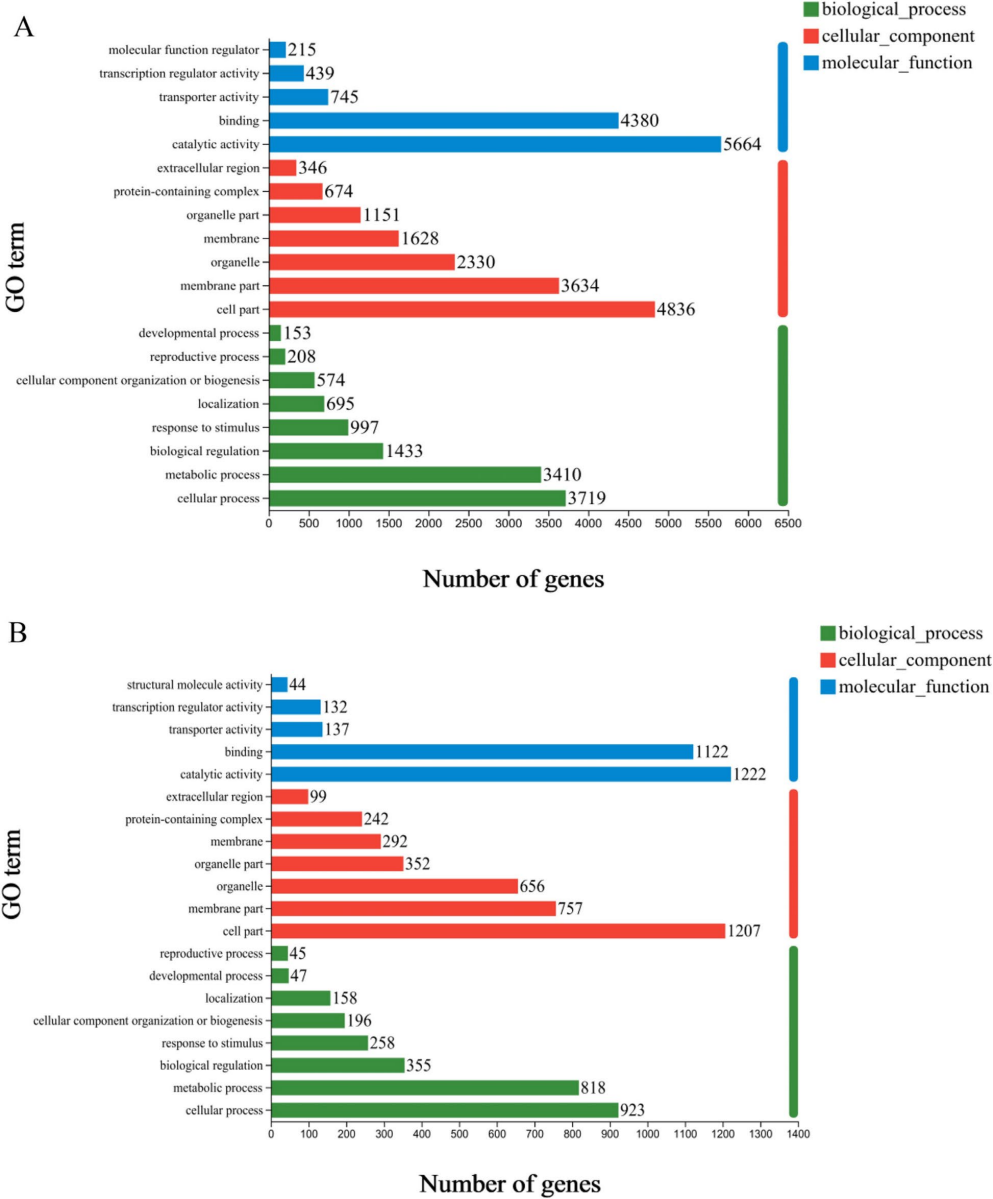
GO functional annotation of differentially expressed genes in LM18 and HL (**A**: GO functional annotation for LM18; **B**: GO functional annotation for HL)

The DEGs of LM18 and HL were subjected to gene function annotation to characterize their biological functions (Table 1). The results revealed that 9,301 and 2,223 DEGs in LM18 and HL, respectively, were annotated in the GO database. Functional annotation revealed that the number of DEGs enriched in six subcategories, including cellular process, was significantly higher in LM18 and HL (Fig. 4A and B). GO enrichment analysis revealed that 65 DEGs in LM18 were significantly enriched in the GO term “response to salt stress(GO:0009651)” (*P* < 0.05), and 6 DEGs were significantly enriched in the “positive regulation of response to salt stress(GO:0043141)” (*P* < 0.05). Both of these GO terms are directly related to the response to salt-alkali stress. All genes enriched in these two GO terms were unique to LM18. In addition, 265, 154, 116, 70, and 51 DEGs in LM18 were significantly enriched in the GO terms “response to abiotic stimulus (GO:0009628)”, “response to external stimulus (GO:0009605)”, “response to oxidative stress (GO:0006979)”, “response to osmotic stress (GO:0006970)”, and “regulation of response to stress (GO:0080134)” (*P* < 0.05)(Additional file 6: Table S4). For HL, 86 and 21 DEGs were significantly enriched in the GO terms response to abiotic stimulus (GO:0009628) and regulation of response to stress (GO:0080134) (*P* < 0.05) (Additional file 7: Table S5).

**Table 1:** Functional annotation and enrichment results of DEGs

Variety (line)	Number of differentially expressed genes	Number of differentially expressed genes annotated	Databases					
			GO	KEGG	COG	Nr	Swiss-Prot	Pfam
LM18	10,289	10,279	9301	2708	9727	10,279	8919	9008
	9501*	9493*	8586*	2474*	8979*	9493*	8246*	8326*
HL	2478	2467	2223	664	2317	2467	2117	2119
	1690*	1681*	1509*	430*	1573*	1681*	1440*	1436*

*Databases:* The number of differentially expressed genes of LM18 and HL in response to saline-alkaline, annotated to the GO, KEGG, COG, Nr, Swiss-Prot and Pfam databases

*: the related data of DEGs only belonged to LM18 and HL

**Fig. 5:**
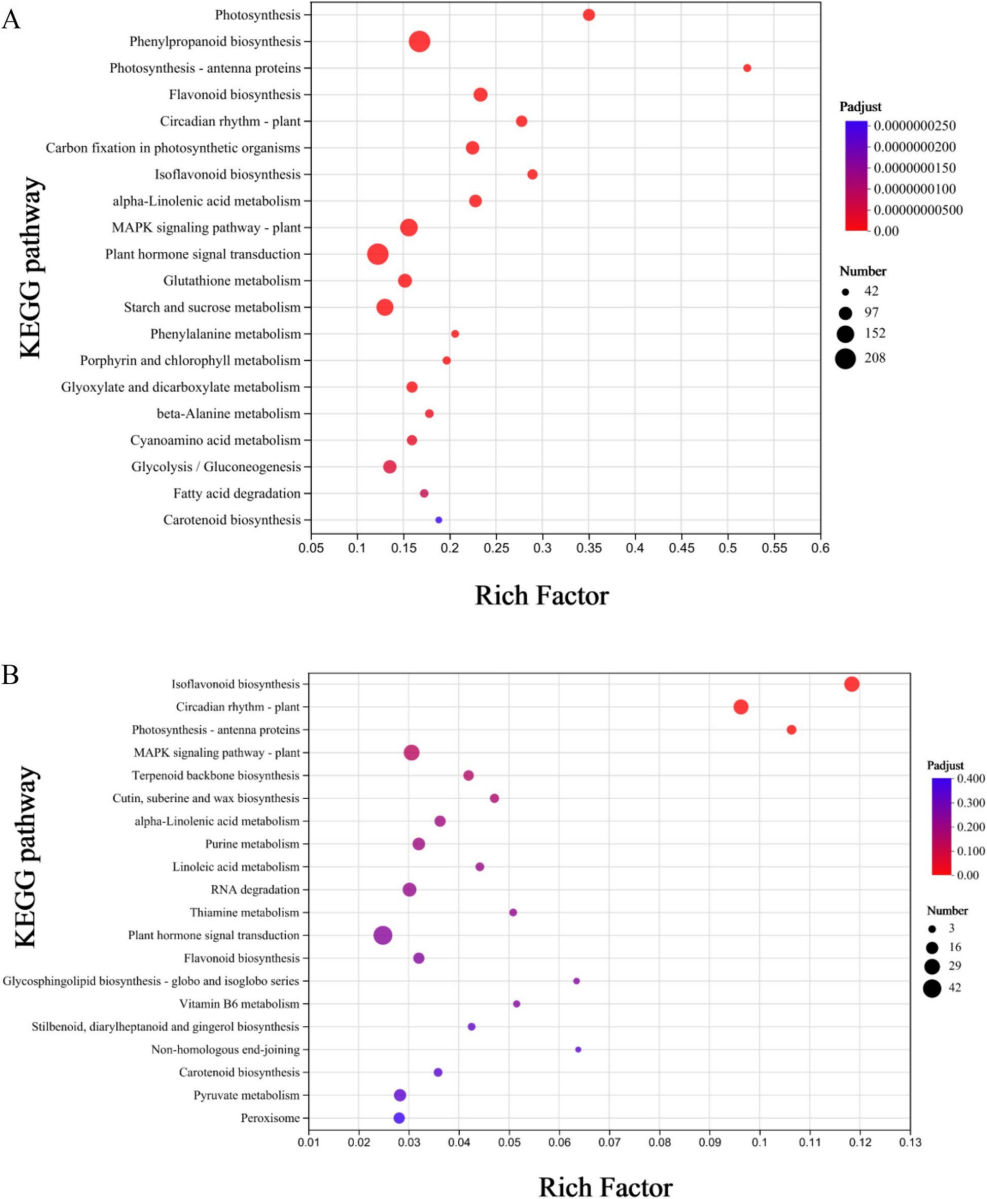
KEGG enrichment analysis of differentially expressed genes in LM18 and HL (**A**: KEGG functional enrichment for LM18; **B**: KEGG functional enrichment for HL. The vertical axis represents the pathway name; the horizontal axis represents the Rich Factor; the larger the Rich Factor, the greater the degree of enrichment; The size of the dot indicates the number of genes in this pathway, while the color of the dot corresponds to different *p*-adjust ranges). Note: The y-axis represents the pathway names, while the x-axis indicates the Rich Factor. A higher Rich Factor reflects a greater degree of pathway enrichment. The same applies to subsequent figures

**Fig. 6:**
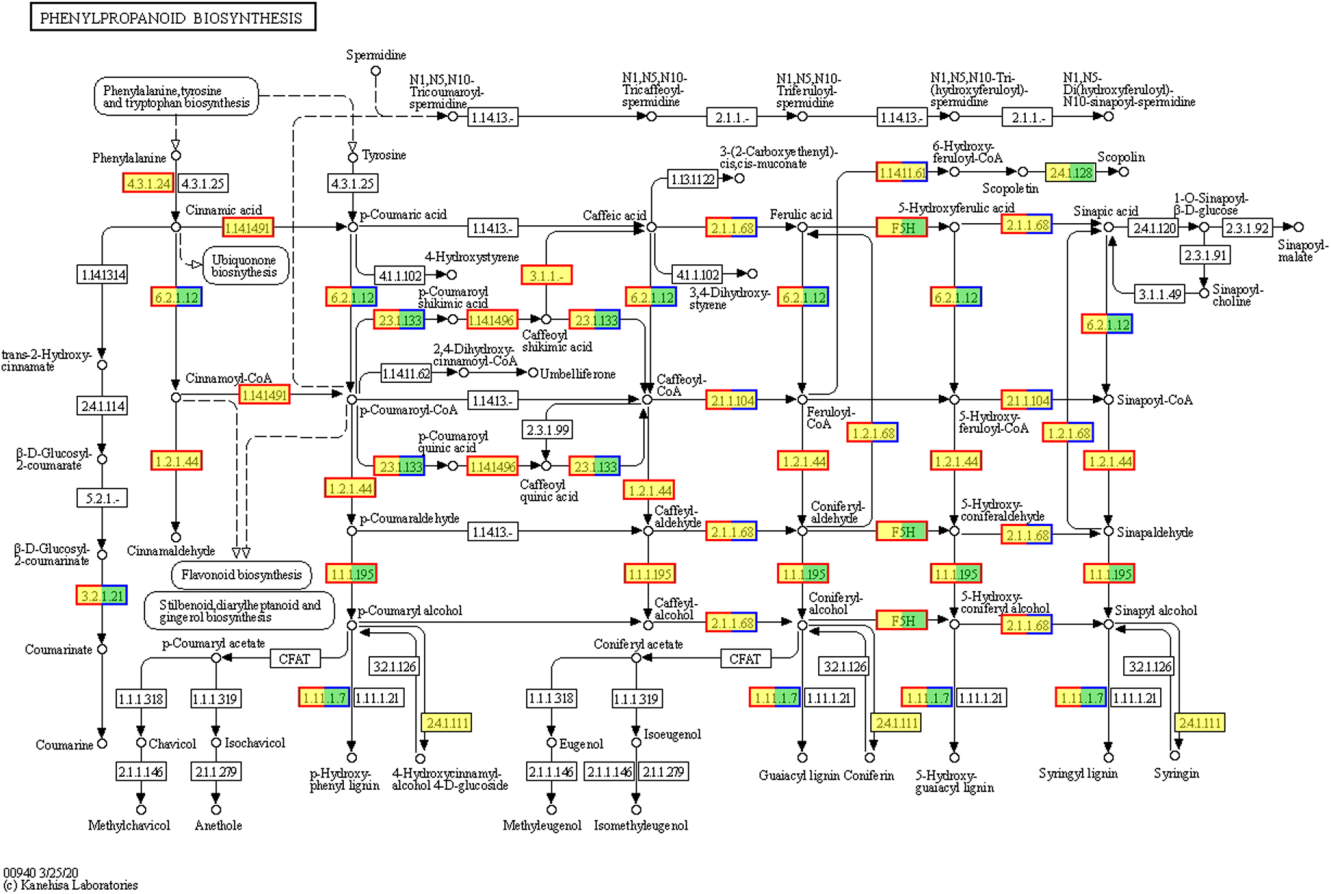
Phenylpropanoid biosynthesis pathway of DEGs in LM18. Note: Red indicates upregulated genes, while blue represents downregulated genes. This color scheme (red for upregulation and blue for downregulation) is consistently applied throughout all pathway figures

**Fig. 7:**
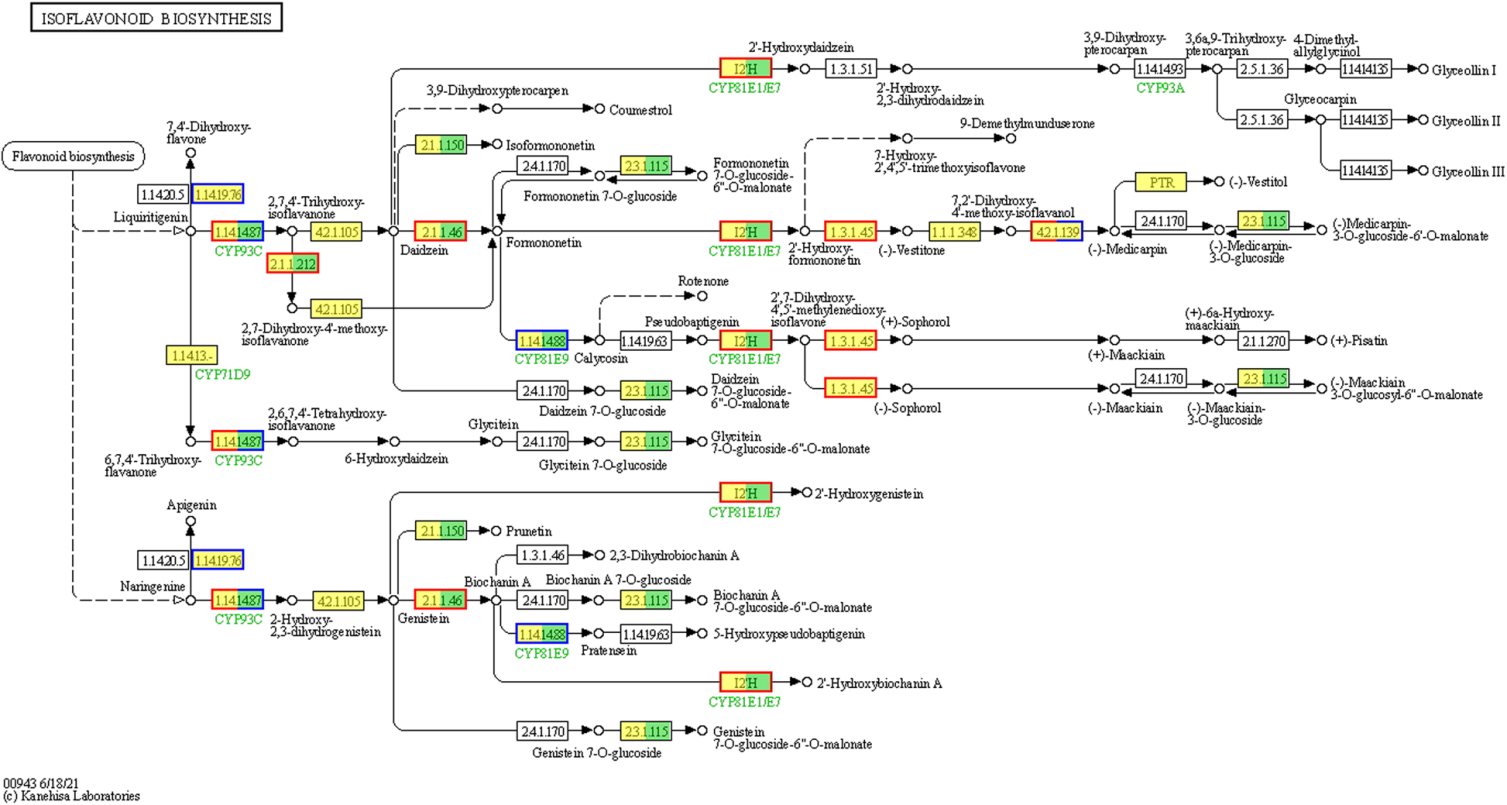
Isoflavonoid biosynthesis pathway of DEGs in HL

Gene products function together to perform critical biological functions within an organism. KEGG pathway annotation analysis of DEGs offers valuable insights into the biological functions of these genes. The KEGG database serves as an essential public resource for pathway information. KEGG pathway analysis of the DEGs in the two *M. falcata* varieties revealed that 2,708 DEGs in LM18 were annotated and significantly enriched in 56 pathways (*P* < 0.05) (Additional file 8: Table S6). The pathway with the highest number of DEG participants in LM18 was the “Phenylpropanoid biosynthesis pathway (map00940)”, involving 208 DEGs (Fig. 5A), most of which were upregulated (Fig. 6). Notably, 164 DEGs were consistently upregulated across all salt-alkali treatment time points in LM18(Additional file 9: Table S7). This pathway was uniquely enriched by DEGs specific to LM18, suggesting that it plays a vital role in the response to salt-alkali stress in this variety. Other significantly enriched pathways in LM18 included “Plant hormone signal transduction (map04075)” and “Plant-pathogen interaction (map04626)”, which further support the involvement of various regulatory and defense mechanisms in LM18's stress response. In contrast, a total of 664 DEGs were annotated in HL, with significant enrichment observed in only three pathways (*P* < 0.05) (Additional file 10: Table S8). Among these pathways, the one with the highest DEG participation was the “Isoflavonoid biosynthesis pathway (map00943)”, which included 27 DEGs (Fig. 5B),the majority of these DEGs were upregulated under salt-alkali stress conditions (Fig. 7). Of these, 19 DEGs were consistently upregulated across all salt-alkali treatment time points in HL (Additional file 11: Table S9). The other two enriched pathways in HL were “Circadian rhythm—plant (map04712)” and “Photosynthesis—antenna proteins (map00196)”.

**Fig. 8:**
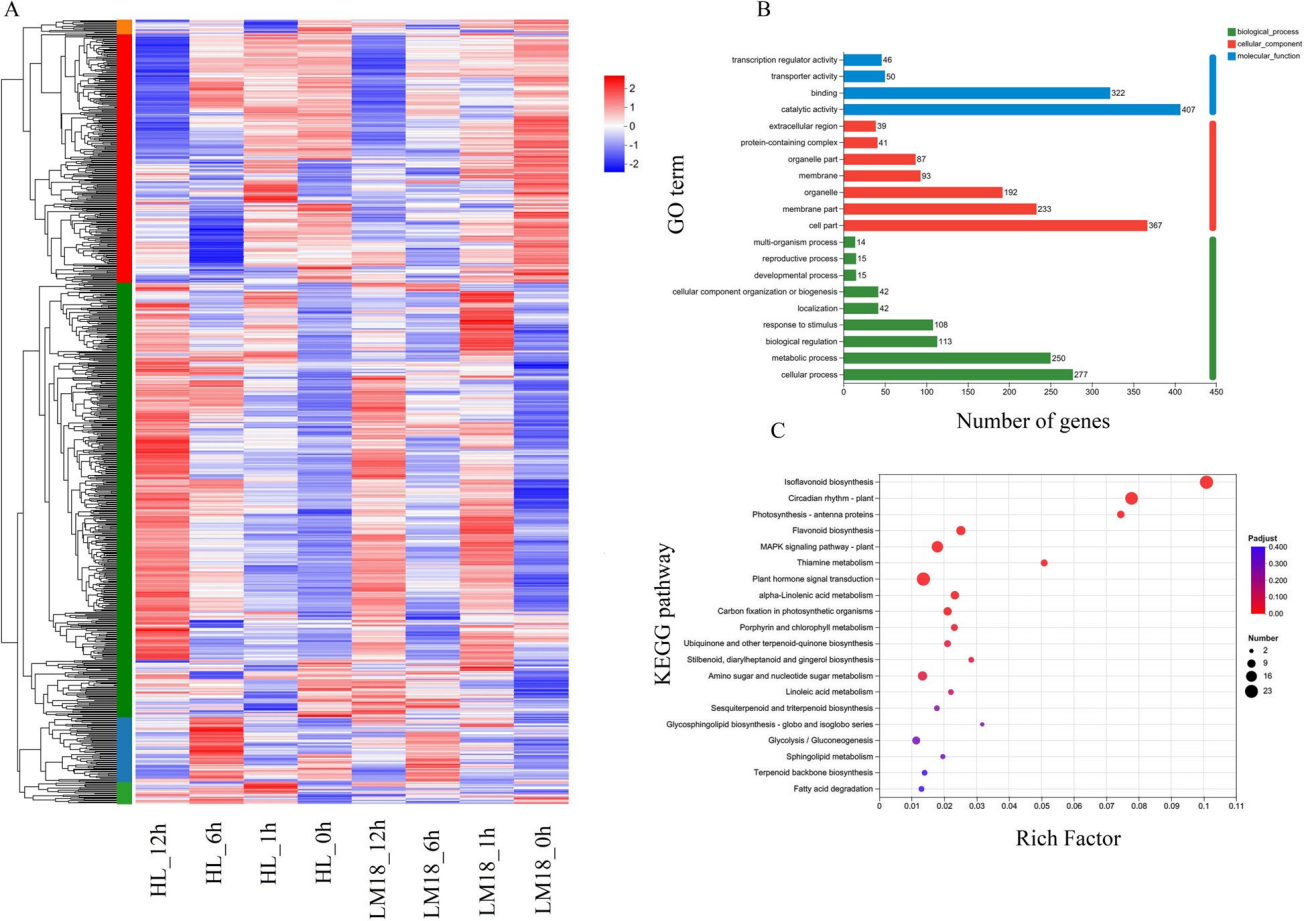
Differentially co-expressed gene analysis of LM18 and HL (**A**: Clustering heatmap of differentially co-expressed genes based on TPM values; **B**: GO functional annotation of differentially co-expressed genes; **C**: KEGG enrichment analysis of differentially co-expressed genes)

Expression pattern clustering was performed on 788 differentially co-expressed genes (shared between LM18 and HL), resulting in five distinct clusters. Among them, Cluster 3 contained the largest number of genes, which exhibited a significant upregulation at the late stage (12 h) of salt-alkali stress in both LM18 and HL, compared to the control condition (0 h) (Fig. 8A). Based on Gene Ontology (GO) functional annotation, a higher number of differentially co-expressed genes were found in the subcategories of *catalytic activity, binding, cell part, membrane part, cellular process,* and *metabolic process* (Fig. 8B). GO enrichment analysis revealed that 107, 45, and 40 differentially co-expressed genes were significantly enriched in the biological processes “*response to stimulus* (GO:0050896)”, “*defense response* (GO:0006952)”, and “*response to abiotic stimulus* (GO:0009628)”, respectively (*P* < 0.05) (Additional file 12: Table S10). KEGG enrichment analysis identified 23, 23, and 21 differentially co-expressed genes that were significantly enriched in the metabolic pathways “*Isoflavonoid biosynthesis* (map00943)”, “*Plant hormone signal transduction* (map04075)”, and “*Circadian rhythm – plant* (map04712)” (Fig. 8C). Notably, 13 differentially co-expressed genes that were significantly enriched in the “*Isoflavonoid biosynthesis* (map00943)” pathway were upregulated across all salt-alkali treatment time points. Similarly, 10 differentially co-expressed genes in the “*Plant hormone signal transduction* (map04075)” pathway showed upregulation, and 7 differentially co-expressed genes in the “*Circadian rhythm – plant* (map04712)”pathway were upregulated (Additional file 13: Table S11).

### Physiological responses of LM18 and HL under salt-alkali stress

**Fig. 9:**
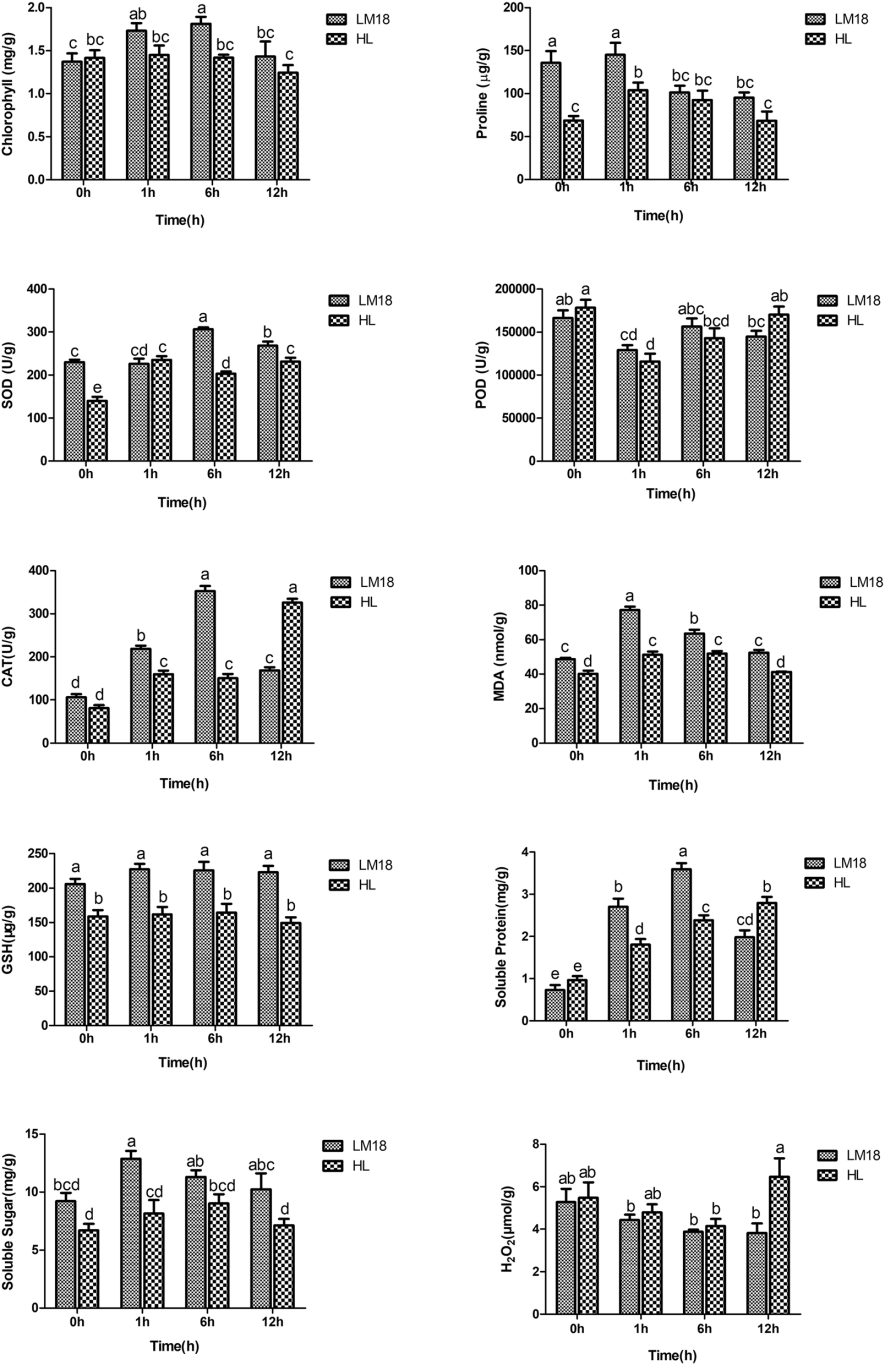
Measurement of physiological indicators in LM18 and HL under salt-alkali stress

Salt-alkali stress commonly induces negative impacts on plant photosynthesis and leaf MDA levels, triggering energy buildup and consequent generation of ROS [44]. In this study, GO enrichment analysis revealed that the DEGs in *M.falcata* were significantly enriched in the GO terms “response to osmotic stress”, “intramolecular oxidoreductase activity”, and “antioxidant activity” at different time points under salt-alkali stress. Most of the DEGs enriched in these three GO terms were upregulated. Under salt-alkali stress, plants actively accumulate osmotic regulators to maintain intracellular osmotic pressure, prevent water loss, and continuously absorb water from the external environment, thereby sustaining normal growth and development. Additionally, plants enhance antioxidant enzyme activity to resist oxidative damage, thereby improving their salt-alkali tolerance. Therefore, the contents of chlorophyll, proline, SOD, POD, CAT, GSH, MDA, H_2_O_2_, Soluble Sugar and Soluble Protein were measured under salt-alkali stress. As illustrated in Fig. 9, under salt-alkali stress, the chlorophyll content in LM18 significantly increased compared to the control (0 h), reaching a maximum at 6 h, while the change in HL was not significant. The proline content in both LM18 and HL initially increased and then decreased compared to the control, peaking at 1 h. The SOD content in LM18 and HL showed an increasing trend, with LM18 peaking at 6 h and HL at 1 h. The POD content in LM18 and HL initially decreased and then increased under salt-alkali treatment, showing a decrease at all time points compared to the control. The CAT content in LM18 and HL significantly increased, with LM18 peaking at 6 h and HL at 12 h. The MDA content in LM18 and HL significantly increased at 1 h under salt-alkali treatment compared to the control, followed by a gradual decrease. The GSH content in LM18 was consistently higher than in HL at all salt-alkali treatment time points. Soluble Protein content in LM18 initially increased and then decreased under salt-alkali stress, peaking at 6 h, whereas HL showed a continuous increase, reaching its highest level at 12 h. Both LM18 and HL exhibited a similar trend in Soluble Sugar content, with levels increasing initially and then decreasing. LM18 had higher Soluble Sugar content at all treatment time points than HL. The H_2_O_2_ content in LM18 gradually decreased under salt-alkali stress, while HL exhibited a decrease followed by an increase, reaching the highest level at 12 h.

PCA and membership function analysis were conducted, and the results in Table 2 show that the cumulative contribution rate of the first two principal components is 74.242%, indicating that these two components effectively summarize the comprehensive indicators for evaluating salt-alkali tolerance in the two alfalfa varieties (LM18 and HL). Principal component 1 (PC1) has an eigenvalue of 5.128 and a contribution rate of 51.276%. The higher coefficients for Soluble Protein (0.899) and MDA (0.866) in PC1 suggest that these indicators play a major role in evaluating salt-alkali tolerance. Principal component 2 (PC2) has an eigenvalue of 2.297 and a contribution rate of 22.966%. In PC2, Proline (0.889) and Chlorophyll (0.365) exhibited higher coefficients, indicating their significant contribution to assessing tolerance. The higher coefficients of Soluble Protein, MDA, Proline, and Chlorophyll in both principal components reflect changes in osmoregulation and cell membrane permeability in alfalfa. Based on these findings, Soluble Protein, MDA, Proline, and Chlorophyll were selected for membership function analysis. The results of the membership function analysis under salt-alkali stress (Table 3) indicate that LM18 exhibits higher salt-alkali tolerance than HL.

**Table 2:** Coefficients and contribution rates of comprehensive indicators

Physiological indicators	PCA1	PCA2
Chlorophll	0.581	0.365
Proline	0.244	0.889
SOD	0.771	-0.571
POD	-0.778	-0.462
CAT	0.756	-0.608
MDA Soluble Sugar Soluble Protein GSH H_2_O_2_	0.866 0.742 0.899 0.648 -0.655	0.442 0.269 -0.416 0.007 0.156
Characteristic value	5.128	2.297
Contribution rate /%	51.276	22.966
Cumulative contribution rate /%	51.276	74.242

Characteristic value: The variance influence value of the principal component of PCA1 or PCA2

Contribution rate /%: The proportion of information extracted by principal component PCA1 or PCA2 to the total information

Cumulative contribution rate /%: The sum of the contribution rate of PCA1 and PCA2

Note: The physiological index coefficients were derived through principal component analysis (PCA) of physiological parameter data using SPSS software. The component matrix (Component Matrix) was used to obtain factor loadings, which were then divided by the square root of the corresponding principal component eigenvalues to calculate the physiological index coefficients. In PCA analysis, these coefficients represent the contribution of each physiological parameter to the principal components. When evaluating salt-alkali tolerance in *M. falcata*, the physiological index coefficients provide a quantitative measure of their influence on the comprehensive salt-alkali tolerance principal component

**Table 3:** Membership function analysis results for *M.falcata* under salt-alkali stress

Variety (line)	Each index membership function value				Average	Ranking
	MDA	Chlorophyll	Soluble Protein	Proline		
LM18	0.87	0.60	0.53	0.66	0.67	1
HL	0.26	0.24	0.44	0.19	0.28	2

### WGCNA and identification of key genes

**Fig. 10:**
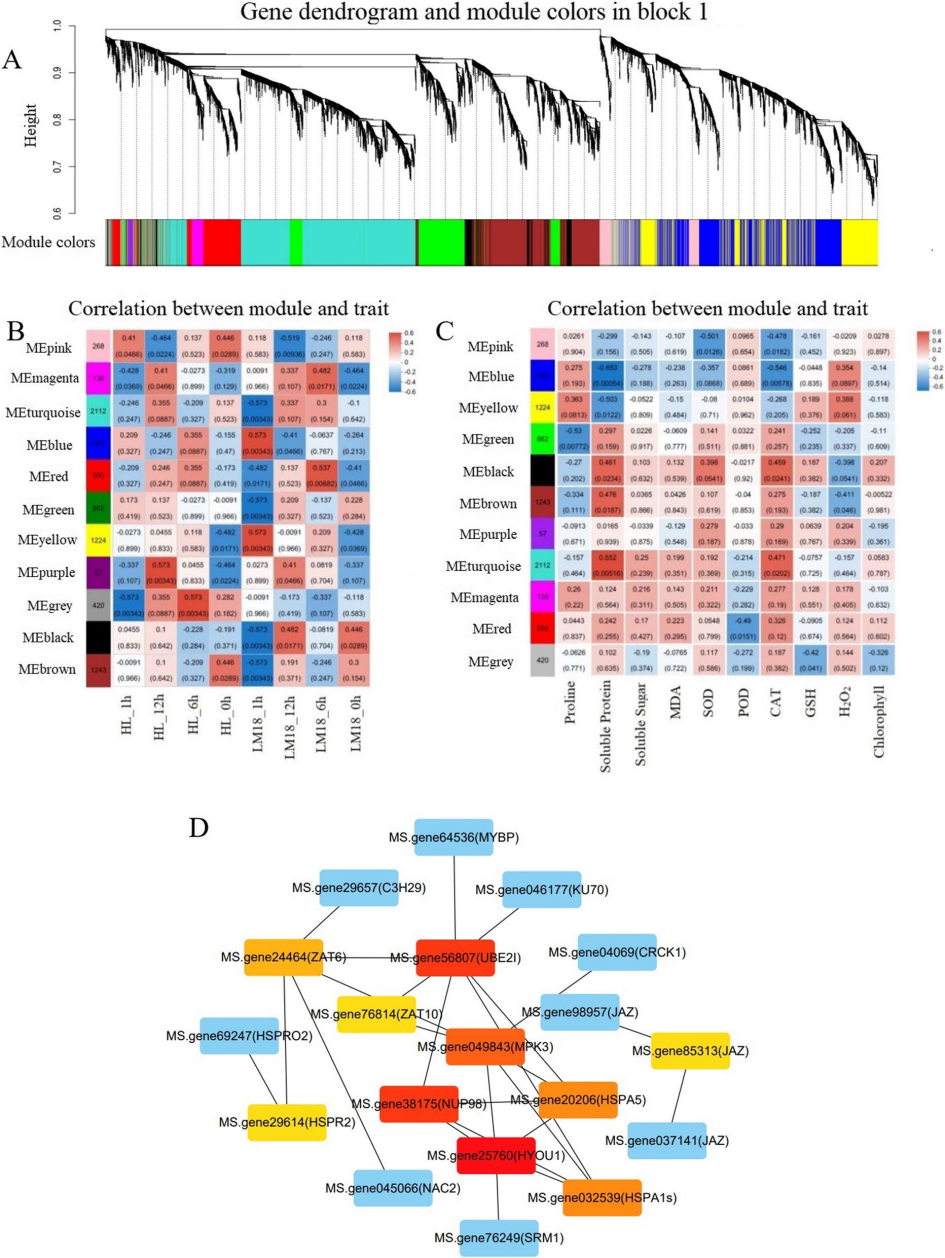
Gene clustering tree and module, module-sample, and module-physiological indicator association, and gene co-expression network in the MEturquoise module (**A**: Clustering tree and modules; **B**: Module-Sample association; **C**: Module-Physiological indicator association; **D**: Gene co-expression network in the MEturquoise module)

Then, WGCNA analysis was implemented on 11,979 genes from LM18 and HL to identify different co-expression modules in *M.falcata* under salt-alkali stress. The minimum number of genes per module was set to 30, with a soft threshold power β of 9 and a module merging threshold of 0.85, resulting in 11 distinct gene modules represented by different colors (Fig. 10A). Among these, the MEturquoise module contained the highest number of DEGs (2,112) related to *M. falcata* samples and physiological indicators under salt-alkali treatment (Fig. 10B, C). Meanwhile, the MEturquoise module exhibited strong correlations with most physiological traits, suggesting that it may play a pivotal role in the salt-alkali tolerance of *M. falcate.* Therefore, the MEturquoise module was selected as the target gene module for further analysis. GO enrichment analysis revealed that 182 DEGs in the MEturquoise module were significantly associated with stress response. KEGG enrichment analysis showed that the 182 DEGs in the MEturquoise module were involved in 20 metabolic pathways, with significant enrichment in the following four pathways: “*Isoflavonoid biosynthesis”*, “*Plant hormone signal transduction”*, “*Amino sugar and nucleotide sugar metabolism”*, and “*Protein processing in the endoplasmic reticulum”* (Additional file 14: Table S12). A protein–protein interaction (PPI) network was constructed for the MEturquoise module using an MCODE score > 2 (Fig. 10D). The kME value of each gene within the module was calculated, identifying 19 hub genes. Among these, *MS.gene64536* (MYBP) and *MS.gene049843*(MPK3) were related to “positive regulation of response to salt stress” and *MS.gene76249*(SRM1) was associated with “response to salt” suggesting that these genes may serve as core genes in *M.falcata*'s response to salt-alkali stress. In the salt-alkali tolerant LM18 *M.falcata*, *MS.gene64536* (MYBP)*, MS.gene76249* (SRM1) and *MS.gene049843* (MPK3) were upregulated at all time points under salt-alkali treatment (Additional file 15: Table S13). Notably, *MS.gene049843* (MPK3) was significantly enriched in the “MAPK signaling pathway – plant” and “Plant-pathogen interaction” pathways. Pfam annotation revealed that *MS.gene64536* (MYBP)*, MS.gene76249* (SRM1) *and MS.gene049843* (MPK3) were annotated with domains including Protein kinase domain, Protein tyrosine and serine/threonine kinase, Lipopolysaccharide kinase, ABC1 atypical kinase-like domain, and Myb-like DNA-binding domain.

### Identification of TFs responsive to salt-alkali stress

**Fig. 11:**
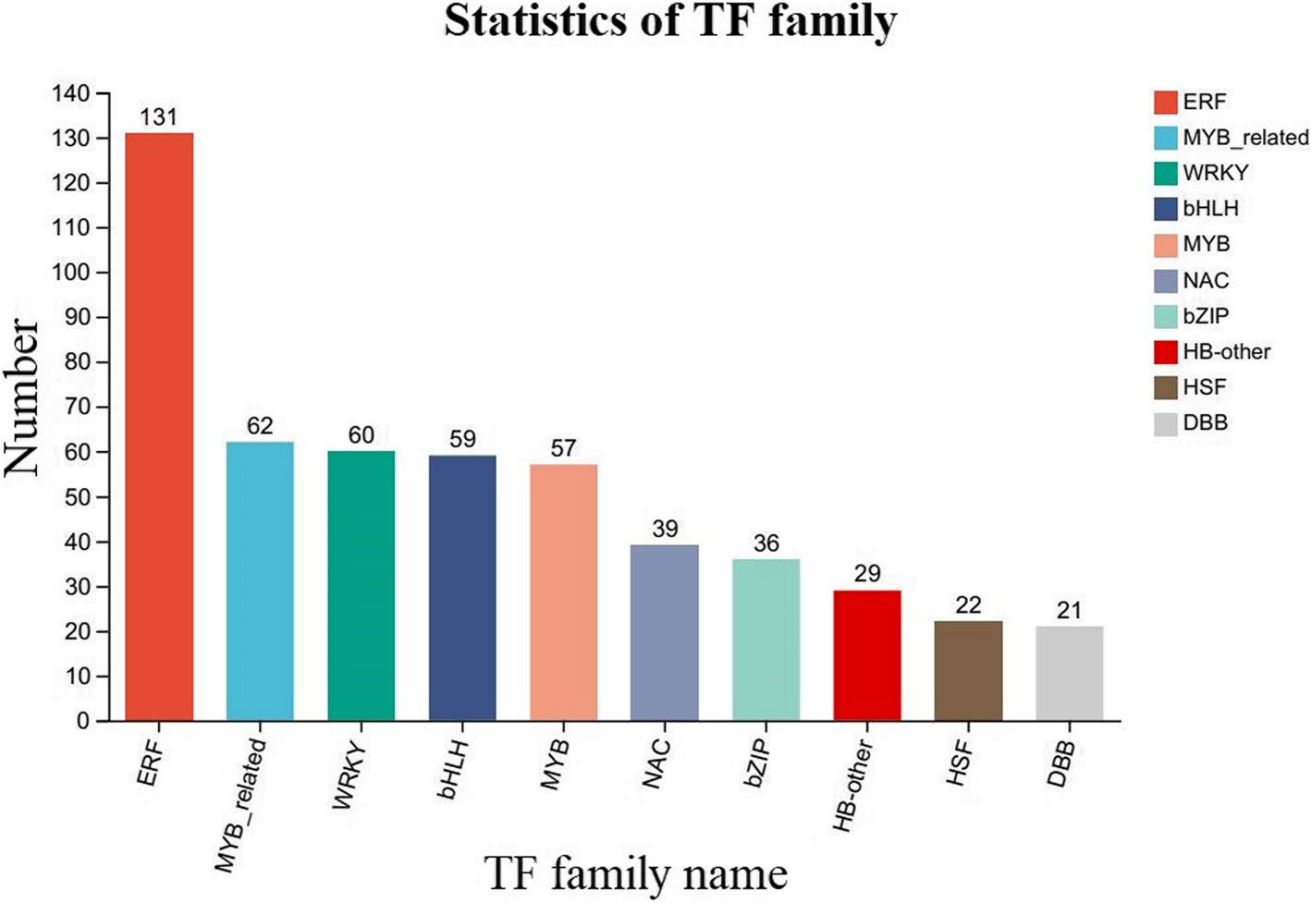
Prediction of transcription factors among differentially expressed genes in LM18 and HL

In plant biology, several TF families are pivotal in mediating responses to diverse environmental stresses, including salt-alkali stress [45]. This study aimed to identify and characterize the TFs involved in plant responses to these stresses, focusing on understanding their collective and individual roles. Among the 11,979 DEGs in LM18 and HL, members of 42 TF families were identified. The ten TF families with the highest number of predicted TFs were ERF (131), MYB_related (62), WRKY (60), bHLH (59), MYB (57), NAC (39), bZIP (36), HB-other (29), HSF (22), and DBB (21) (Fig. 11). Following salt-alkali stress, 545 TF-encoding genes were identified in LM18, while 160 were identified in HL, with LM18 showing a significantly higher number of transcription factor-encoding genes than HL. The WGCNA-identified *MS.gene64536* gene belongs to the MYB-type transcription factor family. Among the 9,501 DEGs unique to LM18 in response to salt-alkali stress, 489 transcription factor-encoding genes belonging to 40 different TF families were predicted.

**Fig. 12:**
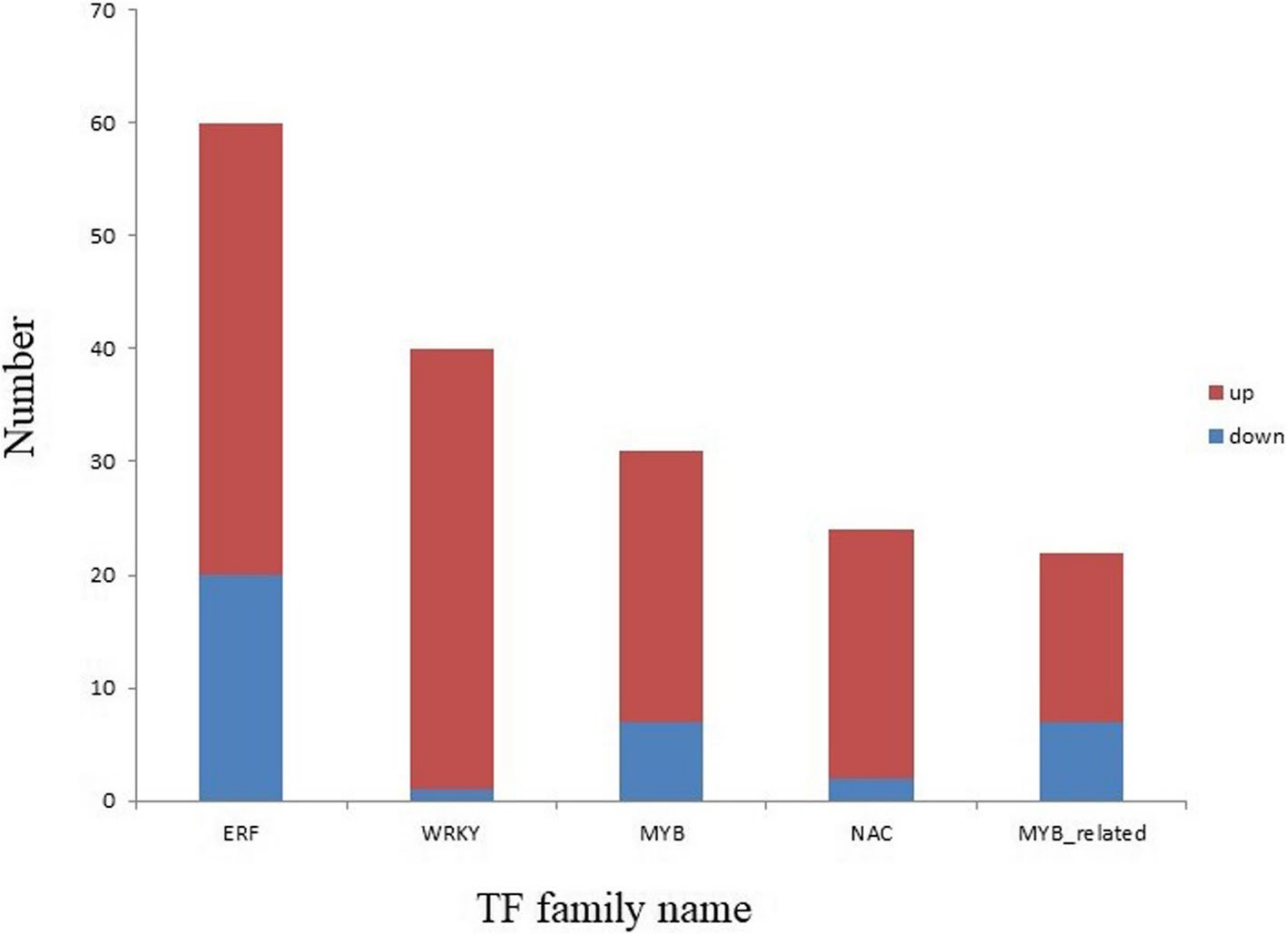
Prediction of transcription factors among LM18-specific differentially expressed genes under 12 h of salt-alkali stress

Furthermore, to identify TFs that are strongly induced by salt-alkali stress and to explore the fundamental molecular mechanisms of the stress response, we further analyzed the TFs unique to the salt-alkali tolerant LM18, specifically those induced after 12 h of salt-alkali stress. The results confirmed that the expression patterns of DEGs in the five most significantly affected TF families (ERF, WRKY, MYB, NAC, and MYB_related) varied. In general, the number of upregulated transcription factor genes was greater than that of downregulated genes (Fig. 12).

### qRT-PCR validation of gene expression

**Fig. 13:**
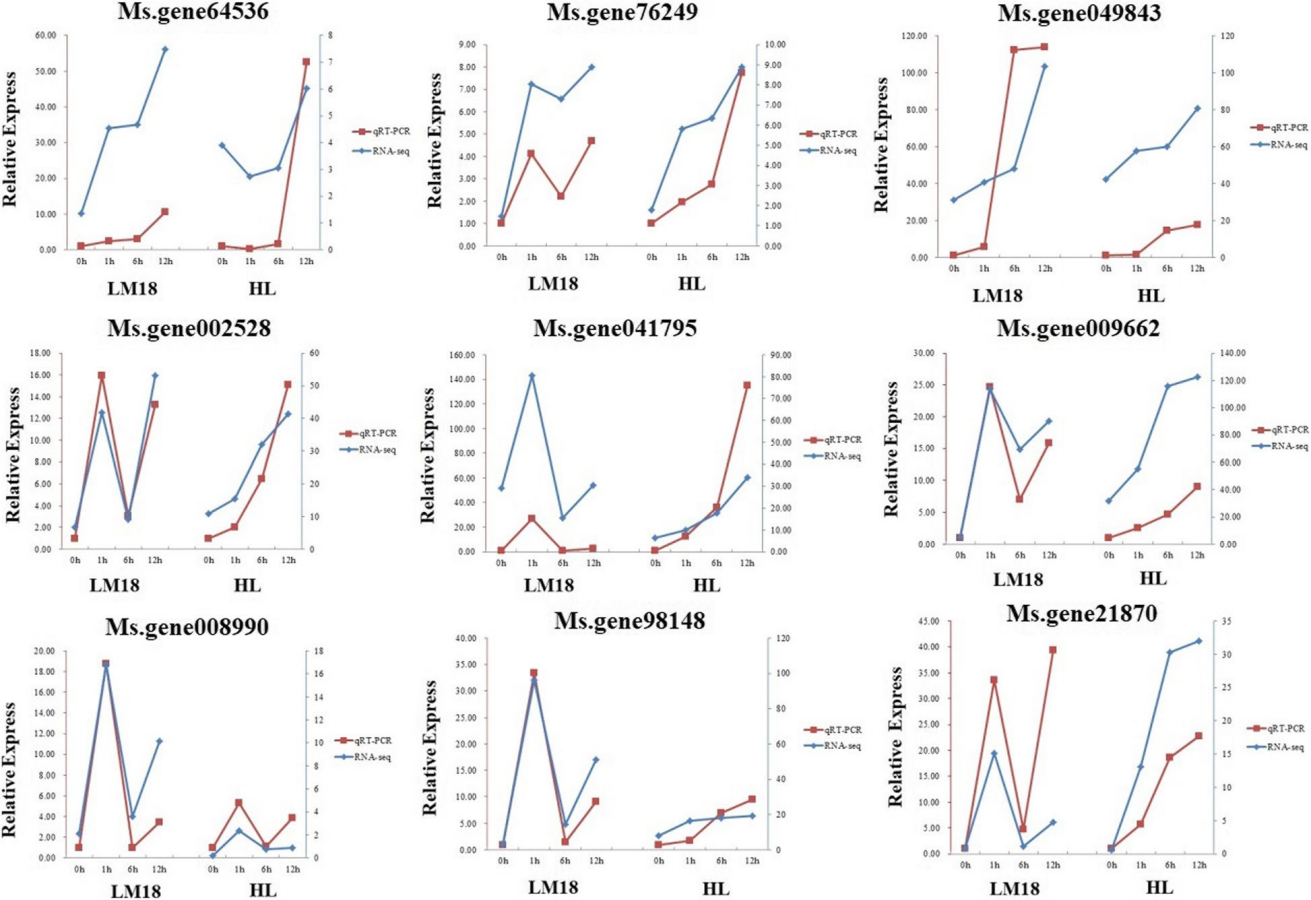
Relative expression levels of nine differentially expressed genes in qRT-PCR analysis. The red line represents the qRT-PCR validation results, while the blue line represents the sequencing results. No treatment (0 h) in the RNA-seq and qPCR results was normalized as “1”

Subsequently, our RNA-seq data was further validated via qRT-PCR validation on the core genes *MS.gene64536* (MYBP), *MS.gene76249* (SRM1) and *MS.gene049843* (MPK3), as well as six randomly selected genes from the 788 differentially co-expressed genes in both LM18 and HL. These six genes included those with transcription factor activity (*Ms.gene002528*), calcium-binding protein (*Ms.gene041795*), oxidoreductase activity (*Ms.gene009662*), plant signal transduction (*Ms.gene008990*), and ligase and transferase activity (*Ms.gene98148* and *Ms.gene21870*). The expression trends of these nine genes at different treatment points were generally consistent with the expression trends observed in the RNA sequencing data, indicating the reliability of the gene expression values obtained in our experiments (Fig. 13).

## Discussion

Plants frequently encounter salt-alkali stress during their growth, which necessitates adaptive strategies to withstand such environmental stresses. At the molecular level, factors such as the genome, transcriptome, proteome, and metabolism play key roles in determining a plant’s tolerance to abiotic stress. Transcriptomic studies offer detailed insights into mRNA expression and play a crucial role in identifying genes involved in stress adaptation [46, 47]. Numerous genes responsive to salt-alkali conditions have been cataloged across diverse studies [48–50]. Research in *Arabidopsis thaliana* and rice shows that the abundance of transcripts for many genes changes in response to abiotic stresses, such as high salinity, cold, and drought [51, 52]. A deeper understanding of these gene networks and their underlying mechanisms is essential for developing improved salt-alkali tolerance in crop breeding programs. In this study, many DEGs were identified in both LM18 and HL within 12 h of salt-alkali treatment, which helped the plants adapt to the salt-alkali environment. KEGG analysis showed that the DEGs in both LM18 and HL were significantly enriched in the “Photosynthesis—antenna proteins” pathway, indicating that salt-alkali stress increases the expression levels of genes involved in photosynthesis, which helps stabilize the photosystem and enhances plant tolerance [53, 54]. Furthermore, both LM18 and HL showed significant enrichment in several other pathways, including “Isoflavonoid biosynthesis”,“Flavonoid biosynthesis”, “Carotenoid biosynthesis”, “Circadian rhythm-plant”, “MAPK signaling pathway-plant”, “alpha-Linolenic acid metabolism” and “Plant hormone signal transduction”. However, the number of DEGs in LM18 was significantly higher than in HL, indicating that while LM18 and HL share a certain number of salt-alkali regulatory pathways, the differences in the number of genes involved in regulating different pathways contribute to the variation in salt-alkali tolerance between the two varieties. KEGG pathway analysis of DEGs revealed that most genes related to metabolic processes, such as “Starch and sucrose metabolism”, “Glycolysis / Gluconeogenesis” and “Porphyrin and chlorophyll metabolism” were upregulated in LM18. These are critical signal transduction events triggered by environmental stress, indicating their involvement in salt-alkali stress response [55]. These pathways play crucial roles in plant responses to abiotic stresses such as salt [56], drought [57], and cold [58]. The DEGs in LM18 were significantly enriched in the “phenylpropanoid biosynthesis” pathway, which is involved in the production of flavonoids, phenolics, and other antioxidant compounds. These antioxidants play a vital role in regulating cellular redox balance, maintaining intracellular homeostasis, and enhancing the salt-alkali stress tolerance of *M. falcata*. Additionally, the phenylpropanoid-derived compounds can activate or modulate the expression of salt-alkali stress-related genes, thereby facilitating the adaptation of *M. falcata* to saline-alkali environments. Interestingly, in this study, HL was found to participate in “Stilbenoid, diarylheptanoid, and gingerol biosynthesis” under salt-alkali stress, a pathway essential for plant resistance to diseases and pests [59], indicating that this pathway may be induced by both biotic and abiotic stresses. Plant salt-alkali tolerance is governed by a range of physiological mechanisms, including water and ion balance, photosynthesis, and cellular redox equilibrium, all modulated by gene expression. Photosynthesis is essential for converting solar energy into chemical energy, but salt-alkali stress negatively affects leaf photosynthesis [60, 61]. In salt-treated soybean seedling leaves, genes encoding components of the light and dark reactions of photosynthesis are slightly suppressed or maintained within the first 4 h but are inhibited at later stages (24 h) [62]. In this study, 28 genes involved in the “photosynthesis, dark reaction” function were downregulated in the leaves of LM18 *M. falcata* during the early (1 h), middle (6 h), and late (12 h) stages of salt-alkali stress, indicating that salt-alkali stress inhibits photosynthesis in the leaves at all stages. Under salt-alkali stress, plants often accumulate certain amino acids in large quantities through alterations in amino acid metabolic pathways. This accumulation helps in osmotic regulation and free radical scavenging, maintaining the stability of functional proteins and metabolic enzymes [63, 64]. In this study, DEGs involved in the “L-phenylalanine metabolic process” and “erythrose 4-phosphate/phosphoenolpyruvate family amino acid metabolic process” in LM18 *M. falcata* were upregulated both at the early (1 h) and late (12 h) stages of salt-alkali treatment, suggesting that amino acid metabolism-related genes in LM18 *M. falcata* play a positive regulatory role under salt-alkali stress. In this study, a total of 788 differentially co-expressed genes were identified, which are likely to play a crucial role in the salt-alkali tolerance of *M. falcata*. These findings provide a valuable reference for further exploration of novel mechanisms and pathways underlying salt-alkali tolerance in *M. falcata*. Functional annotation revealed that these DCEGs are associated with "response to stimulus," "response to abiotic stimulus," and "plant hormone signal transduction." The expression of these genes may enable *M. falcata* to perceive salt-alkali stress and efficiently transmit stress signals to the intracellular environment, thereby activating adaptive response mechanisms. Additionally, these genes may regulate various physiological and biochemical processes in *M. falcata*, such as activating the antioxidant system to prevent cellular damage, increasing the synthesis of osmotic regulatory substances to maintain water balance, and preserving normal cellular functions under salt-alkali stress.

Chlorophyll is an essential photosynthetic pigment in plant leaves that provides the necessary materials and energy for plant growth. The content of chlorophyll directly affects the strength of photosynthesis in plants, making it an important indicator of photosynthetic capacity [65]. In this study, under-untreated conditions (0 h), the chlorophyll content in LM18 and HL was similar, with HL showing a slightly higher chlorophyll content than LM18. As the duration of salt-alkali stress increased, the chlorophyll content in LM18 significantly increased, peaking at 6 h before decreasing at 12 h. In contrast, the changes in HL chlorophyll content were less significant, with a slight decrease at 12 h of stress. The chlorophyll content measurements suggest that, under salt-alkali stress, LM18 consistently exhibited higher chlorophyll content compared to HL at all treatment time points, indicating that LM18 has a stronger adaptive ability to salt-alkali stress than HL. Additionally, this finding suggests that salt-alkali stress affects chlorophyll content in plants, and chlorophyll content could serve as an indicator of the plant's salt-alkali tolerance to some extent [15]. Under water stress caused by salt-alkali and other adverse conditions, plants accumulate organic and inorganic solutes to increase solute concentration, thereby lowering cell water potential and maintaining normal cell water content to adapt to the stress. Proline, an osmoprotectant, plays a crucial role in water retention, protecting cell structure, regulating cell pH, and acting as a key marker of stress [66]. In this study, proline content in the leaves of *M. falcata* increased significantly during the early stages of salt-alkali stress (1 h), with the salt-alkali tolerant LM18 showing notably higher proline levels than HL. However, proline content decreased as the stress duration lengthened. Soluble proteins and soluble sugars are key osmoregulatory factors for plants under abiotic stresses such as salt-alkali conditions. They can be directly induced by various environmental factors [67]. In this study, the soluble sugar content in LM18 was consistently higher than that in HL under salt-alkali stress. The soluble protein content in LM18 was also higher than that in HL at 1 h and 6 h of salt-alkali treatment, although it was slightly lower than HL at 12 h. When plants are subjected to stress, excessive ROS are generated, which can oxidize proteins, lipids, and other cellular components, leading to cell death and impairing normal growth [68, 69]. Enzymes such as SOD, POD, CAT, and MDA are critical components of the plant antioxidant system, while glutathione (GSH) functions as a non-enzyme antioxidant. SOD is a key enzyme in the ROS scavenging system, helping plants protect themselves from oxidative damage by neutralizing excess ROS [70]. POD and CAT can remove toxic ROS intermediates in the early stages of stress, playing a protective role for plants [71]. However, in the later stages of stress, POD can promote the generation of ROS, which can be harmful to plants. Waszczak et al. [72] reported increased activities of CAT and SOD in rice leaves under low-phosphorus stress, which is consistent with our findings. As the duration of salt-alkali stress increased, the POD content in both LM18 and HL decreased to varying degrees compared to the control, particularly at 12 h of stress, where LM18 showed lower POD activity than HL. LM18 also showed higher glutathione (GSH) levels under salt-alkali stress compared to HL, while H_2_O_2_ content was lower in LM18 throughout the stress period. These results suggest that LM18 possesses a stronger antioxidant capacity than HL, demonstrating a higher ROS scavenging ability and better regulation of H_2_O_2_ accumulation, which helps mitigate ROS-induced damage in *Medicago falcata* L. [73]. MDA is a product of lipid peroxidation and is commonly used to assess the extent of cell membrane damage under stress [74]. In this study, MDA content in both LM18 and HL initially increased and then decreased with prolonged stress but remained higher than in the control. The MDA content in the salt-alkali tolerant LM18 was higher than in HL under salt-alkali treatment, indicating that salt-alkali stress enhanced lipid peroxidation in *M. falcata*. Under salt-alkali stress, the DEGs associated with chlorophyll biosynthesis in *M. falcata* LM18 were upregulated, suggesting that *M. falcata* can better perceive light signals. This enhanced perception may facilitate signal transduction, promoting chlorophyll synthesis and leading to an increase in chlorophyll content. Furthermore, the upregulation of DEGs may regulate the expression of transporter genes, which facilitate the transport of osmoregulatory substances such as proline from the cytoplasm to the vacuole for storage. This mechanism enhances intracellular proline accumulation, thereby improving osmotic adjustment under salt-alkali stress. Additionally, the upregulation of antioxidant defense-related DEGs may directly enhance the activity of SOD and other antioxidant enzymes, thereby increasing the plant's capacity to scavenge ROS and mitigating oxidative damage.

WGCNA is a systems biology approach widely used to study various biological processes [43], especially for identifying genes associated with specific traits, classifying them into modules, and determining co-expression modules with high biological significance [75]. By analyzing the gene interaction networks within salt-alkali tolerance-related modules, 19 highly connected genes were identified. Previous studies have shown that salt stress can activate the interaction between MPK3 and ARR1/10/12, leading to the phosphorylation of ARR1/10/12 and promoting their degradation under salt stress [76]. MYB proteins are also known to contribute to plant salt tolerance, with many of their downstream targets identified. For example, MYB49 regulates salt tolerance by modulating cuticle formation and antioxidant defense [77]. In this study, GO functional analysis of *MS.gene049843* (MPK3) and *MS.gene64536* (MYBP) revealed their involvement in “positive regulation of response to salt stress” with MYBP being significantly upregulated at all salt-alkali treatment time points in LM18. Meanwhile, *MS.gene76249* (SRM1) was found to have the “response to salt stress” function. KEGG functional annotation revealed that *MS.gene049843* (encoding MPK3) was involved in both the “MAPK signaling pathway—plant” and the “Plant-pathogen interaction” pathways. This suggests that under salt-alkali stress, the *MPK3* gene may be activated via the MAPK signaling cascade, leading to the phosphorylation of downstream target proteins and subsequent regulation of genes associated with salt-alkali tolerance. Although the “Plant-pathogen interaction” pathway is typically associated with defense responses against biotic stress, it may also play a role under abiotic stress conditions such as salt-alkali stress. MPK3 may enhance the plant’s overall defense capacity by phosphorylating proteins involved in defense signaling, thereby contributing to the maintenance of cellular integrity and functionality under stress. Such enhanced defense responses could indirectly improve the salt-alkali tolerance of *M.falcata.* Both *MPK* and *MYB* genes play critical roles in plant growth, development, and stress responses, while SRM1 is a member of the MYB transcription factor family and is widely expressed in plant species. Studies have shown that MPK3 and MPK6 are highly homologous, and as key factors in salt tolerance regulation, they enhance plant salt resistance by phosphorylating the Na + /H + transporter SOS1 [78]. In Arabidopsis, AtSRM1 is an important transcriptional regulator that influences ABA biosynthesis and signaling. Knockout of this gene has been shown to improve seed germination and seedling survival under salt stress [79]. The phosphorylation of MPK genes enhances the DNA-binding capacity of MYB, which is essential for their physiological function in salt stress tolerance [80]. The key genes identified in this study, *MS.gene64536* (MYBP), *MS.gene049843* (MPK3), and *MS.gene76249* (SRM1), involved in salt-alkali stress response in *M. falcata*, warrant further investigation into their interactions. The *MPK* gene can activate the DNA-binding or transcriptional activation ability of MYB through phosphorylation, thereby promoting the expression of downstream salt-alkali stress-related genes, such as those encoding antioxidant enzymes, ultimately enhancing the adaptability of *M. falcata* to salt-alkali stress. Moreover, *MPK* and *MYB* genes may function synergistically within the same signaling pathway, where MPK transmits the stress signal, while MYB binds to the promoter regions of target genes, regulating their expression and collectively enhancing salt stress tolerance in *M. falcata.* Previous studies have reported that the *SRM* gene is induced by ABA (abscisic acid) and regulates the response of poplar (*Populus*) to salt stress [81]. Additionally, the MAPK cascade has been shown to regulate ABA signaling and mediate salt-alkali stress responses in rice (*Oryza sativa*) [82]. Based on these findings, we hypothesize that *SRM* and *MPK* genes may indirectly influence salt-alkali tolerance in *M. falcata* through ABA-mediated signaling pathways. These genes may provide a new theoretical basis and operational components for regulating salt tolerance in *M. falcata*, potentially enriching the molecular regulatory model and signal transduction network for salt-alkali tolerance in this species. Although the biological functions of these three genes require further exploration and validation, the qRT-PCR validation results were consistent with the transcriptome sequencing results, indicating the accuracy of the transcriptome sequencing and the reliability of the WGCNA co-expression network analysis method. In conclusion, this strategy for screening salt-alkali stress-related functional genes is of great significance for research on salt-alkali tolerance in *M. falcata* [83].

TFs are central regulatory elements of gene expression, modulating fundamental functions in plants, including responses to environmental factors and hormones, as well as cell differentiation and organ development [84]. By directly interacting with proteins, TFs regulate gene expression and are critical in governing plant responses to abiotic stress, particularly under salt-alkali conditions. In this study, five TF families most involved in salt-alkali stress responses were identified: ERF, MYB-related, WRKY, bHLH, and MYB. These TFs likely play a defensive role by regulating the expression of downstream genes. ERF is a member of the AP2/ERF superfamily, and it maintains ion and ROS homeostasis by targeting and regulating genes involved in Na + /K + transport and antioxidant enzymes, playing a crucial role in plant defense responses. For instance, the MdERF106 gene in *Musca domestica* L. promotes the expression of MdSOS1, enhancing Na + efflux [85]. ERF TFs play a crucial role in regulating plant salt tolerance and can serve as an important source of salt-tolerant gene resources [86]. Studies have found that the *ERF* gene *PalERF109* in poplar is rapidly induced by salt stress, and overexpression of *PalERF109* enhances salt tolerance by directly activating the expression of high-affinity K^+^ transporter (HKT) genes [87]. Furthermore, the *AP2/ERF* gene *HuERF1* from dragon fruit (*Hylocereus undatus*) has been shown to positively regulate salt tolerance in plants [88]. Additionally, the maize transcription factor ZmEREB20 enhances salt tolerance in transgenic *Arabidopsis* plants [89]. WRKY is one of the largest TF families in plants, involved in abiotic stress responses through plant hormone signaling pathways and ROS scavenging mechanisms [90]. Salt-alkali stress is a widespread form of abiotic stress that severely affects crop yields. WRKY transcription factors play a critical role in regulating plant responses to salt-alkali stress. The regulatory networks formed by WRKY and other genes help maintain plant homeostasis, enabling plants to adapt to salt-alkali environments. Previous studies have shown that overexpression of *ZmWRKY104* in maize (*Zea mays*) positively regulates the expression of *ZmSOD4*, enhancing salt tolerance by alleviating the accumulation of ROS, malondialdehyde (MDA), and increased electrolyte leakage percentage, thereby improving the plant’s salt tolerance [91]. Overexpression of *AtWRKY25* and *AtWRKY33* in *Arabidopsis thaliana* has increased salt tolerance, with 31 and 208 potential ABA-related downstream targets identified, respectively [92]. Members of the bHLH family are critical in various biological processes. Studies have shown that bHLH expression enhances plant tolerance under salt stress by regulating Na + /K + ion transport, ROS clearance, and osmotic adjustment [93]. For example, under salt stress, the expression of the *BvBHLH92* gene in sugar beet is significantly upregulated in both roots and leaves [94]. Additionally, under high salt conditions, the transcription level of *OsbHLH148* in rice rapidly increases [95]. MYB transcription factors play a crucial role in maintaining osmotic potential and ion homeostasis under salt-alkali stress by regulating the composition of plant cell walls and the expression of ion transport proteins, thereby participating in the plant's response to salt-alkali stress. The role of MYB TFs in salt tolerance has been well-studied in *Arabidopsis thaliana*, where the AtMYB49 gene is upregulated under salinity conditions and functions as a key enhancer of the salt stress response [96]. Overexpression of *SlMYB102* in tomato (*Solanum lycopersicum*) significantly increases the transcription levels of genes such as *SlSOS1*, *SlSOS2*, and *SlHAK5*, and by expelling excess Na^+^ from the cytoplasm, compartmentalizing it into vacuoles, and promoting K^+^ absorption, transgenic tomato plants show reduced Na^+^ content and increased K^+^ content in their leaves and roots under salt stress, thus maintaining Na^+^/K^+^ homeostasis and enhancing the salt tolerance of the transgenic plants [97]. Meanwhile, in plants, MYB is primarily involved in inducing the synthesis of secondary metabolites, including anthocyanins, flavonoids, and lignans, which help mitigate oxidative damage by promoting endogenous ROS scavenging [98, 99]. In this study, *MS.gene64536* (*MYBP*), which encodes an MYB TF, was found to be upregulated under salt-alkali stress in LM18. This suggests that *MYBP* may regulate the expression of salt-alkali-responsive genes as a transcriptional regulator. Previous studies have also reported the upregulation of MYB TFs in response to salt stress in alfalfa. For instance, *MsG0280010554.01*, encoding an MYB TF, was upregulated in the leaves of *M.sativa* under salt stress [100], and *MYB4* was highly expressed in ZM-*M.sativa* under similar conditions [101]. These findings imply that MYB TFs may enhance salt-alkali tolerance through conserved mechanisms, such as binding to promoter regions of downstream target genes involved in ion transport, osmotic adjustment, or antioxidant defense. This suggests that MYB TFs play a conserved role in mediating plant responses to salt-alkali stress, highlighting their importance in stress-responsive transcriptional networks. These TF genes were highly enriched in the DEGs under salt-alkali stress, suggesting their collective involvement in the modulation of salt-alkali tolerance in *M. falcata*. In future studies, further investigation into the regulatory mechanisms of transcription factors (e.g., ERF, MYB, WRKY) in the downstream interactions of multiple signaling pathways under salt-alkali stress in *M. falcata* could provide a more precise and efficient approach to enhancing and improving its salt-alkali tolerance. Understanding these mechanisms is of great significance for the breeding of salt-alkali tolerant *M. falcata* varieties, offering valuable insights for the genetic improvement of forage crops adapted to saline-alkali environments.

## Conclusion

In conclusion, this study combined RNA-seq and WGCNA analyses to identify key DEGs related to salt-alkali tolerance in *M. falcata*, including *MS.gene64536* (MYBP), *MS.gene76249*(SRM1), and *MS.gene049843*(MPK3). We also identified the MEturquoise gene module associated with salt-alkali tolerance and pinpointed 42 transcription factor families, with ERF, WRKY, MYB, NAC, and MYB_related being the major regulatory factors involved in salt-alkali stress response. The findings of this study highlight the significant induction of key DEGs and transcription factors under salt-alkali stress, which can be further explored to elucidate their regulatory mechanisms and applied to the improvement of salt-alkali tolerance in *M. falcata*. This research not only provides valuable insights into the key regulatory factors for molecular breeding of salt-alkali tolerance in *M. falcata*, but also offers a fresh perspective on the plant’s adaptation mechanisms to salt-alkali environments. Additionally, it lays the foundation for future studies aimed at improving crop salt-alkali tolerance through genetic engineering technologies. The genes identified in this study may hold significant translational potential for advancing crop breeding programs and developing innovative biotechnological strategies for stress mitigation. Their functional characterization provides a promising foundation for improving salt-alkali tolerance in crops, offering new hope for enhancing agricultural productivity in saline-alkaline soils amid the global expansion of salt-affected land.

## Supplementary Information

Additional file 1: Table S1. qRT-PCR primer of genes selected for RNA-seq data confirmation.

Additional file 2: Table S2. Overview of the quality of the sequence data obtained by RNA-seq sequencing.

Additional file 3: Fig. S1. Correlation analyses of LM18.

Additional file 4: Fig. S2. Correlation analyses of HL.

Additional file 5: Table S3. Summary table of the top 10 up-regulated genes in LM18 and HL.

Additional file 6: Table S4. GO functional annotation of LM18 differentially expressed genes.

Additional file 7: Table S5. GO functional annotation of HL differentially expressed genes.

Additional file 8: Table S6. KEGG functional annotation of LM18 differentially expressed genes.

Additional file 9: Table S7. Expression profiles of DEGs enriched in the “Phenylpropanoid biosynthesis” pathway.

Additional file 10: Table S8. KEGG pathways are significantly enriched in DEGs from HL.

Additional file 11: Table S9. Expression profiles of DEGs enriched in the “Isoflavonoid biosynthesis” pathway.

Additional file 12: Table S10. Co-DEGs GO function annotation.

Additional file 13: Table S11. Co-DEGs involved in major metabolic pathways.

Additional file 14: Table S12. MEturquoise Module gene GO and KEGG function annotation.

Additional file 15: Table S13. Expression profiles of hub genes in the MEturquoise module.

## Data Availability

All relevant supplementary data are provided within this manuscript as Additional files. The RNA-seq data generated in this study were submitted to the NCBI Sequence Read Archive under accession numbers BioProject PRJNA 1164734.

## Abbreviations

DEGs: Differentially expressed genes
GO: Gene Ontology
ROS: Reactive oxygen species
MDA: Malondialdehyde
GSH: Glutathione
TPM: Transcripts per million reads
KEGG: Kyoto Encyclopedia of Genes and Genomes
TBA: Thiobarbituric acid
POD: Peroxidase
SOD: Superoxide dismutase
NBT: Nitroblue tetrazolium
CAT: Catalase
LSD: Least significant difference
PCA: Principal component analysis
WGCNA: Weighted gene co-expression network analysis
