# Supplementary figures and images for: Comparative transcriptome analysis of differentially expressed genes of Medicago falcata L. breeding lines response to saline-alkaline stress
Source: BMC Plant Biol. 2025 May 13;25:623. doi: 10.1186/s12870-025-06599-3 (PMC12070579; doi:10.1186/s12870-025-06599-3)

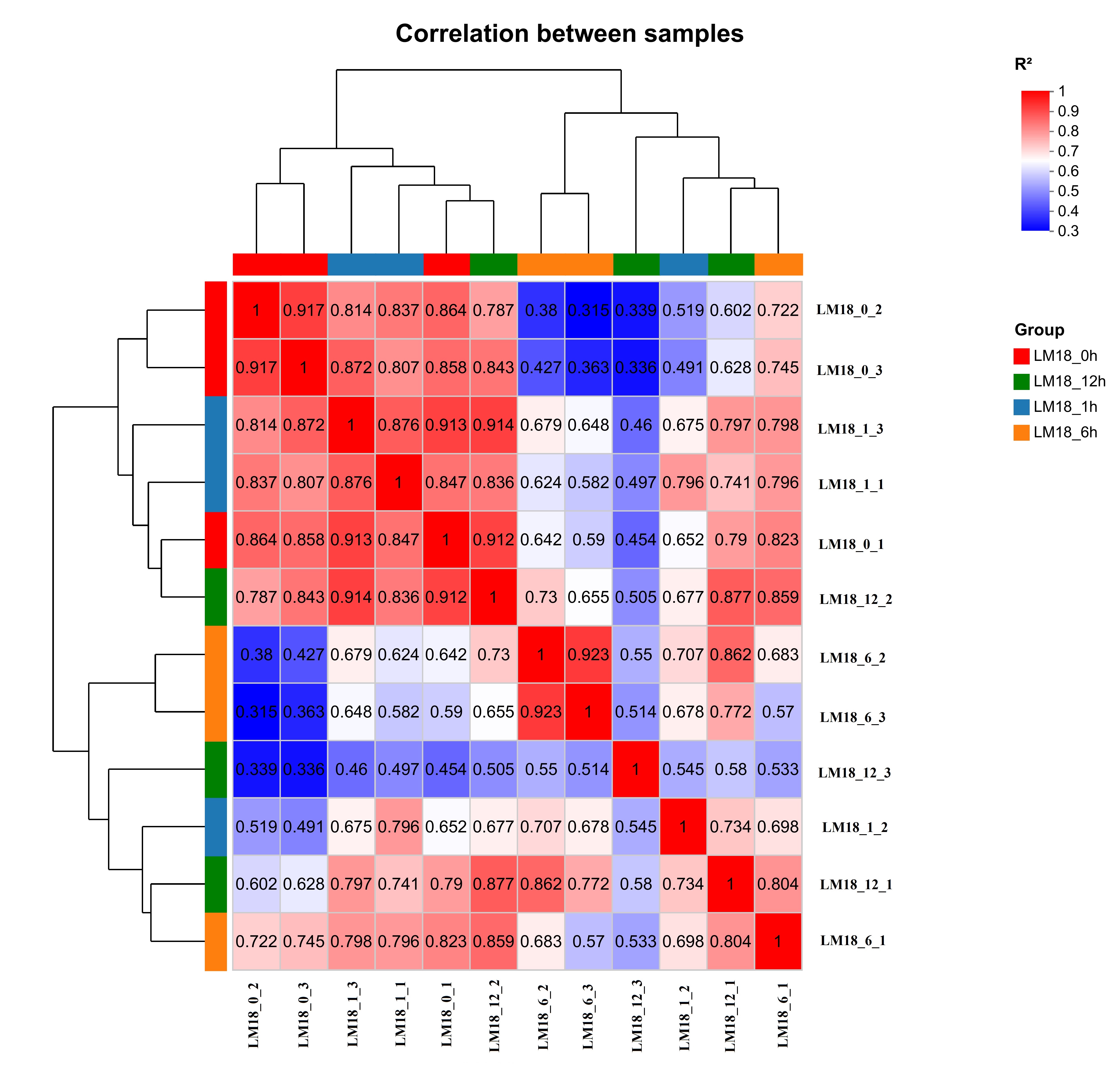

Supplement: Supplementary file 3 — Additional file 3: Fig. S1. Correlation analyses of LM18. [file 12870_2025_6599_MOESM3_ESM.jpg]

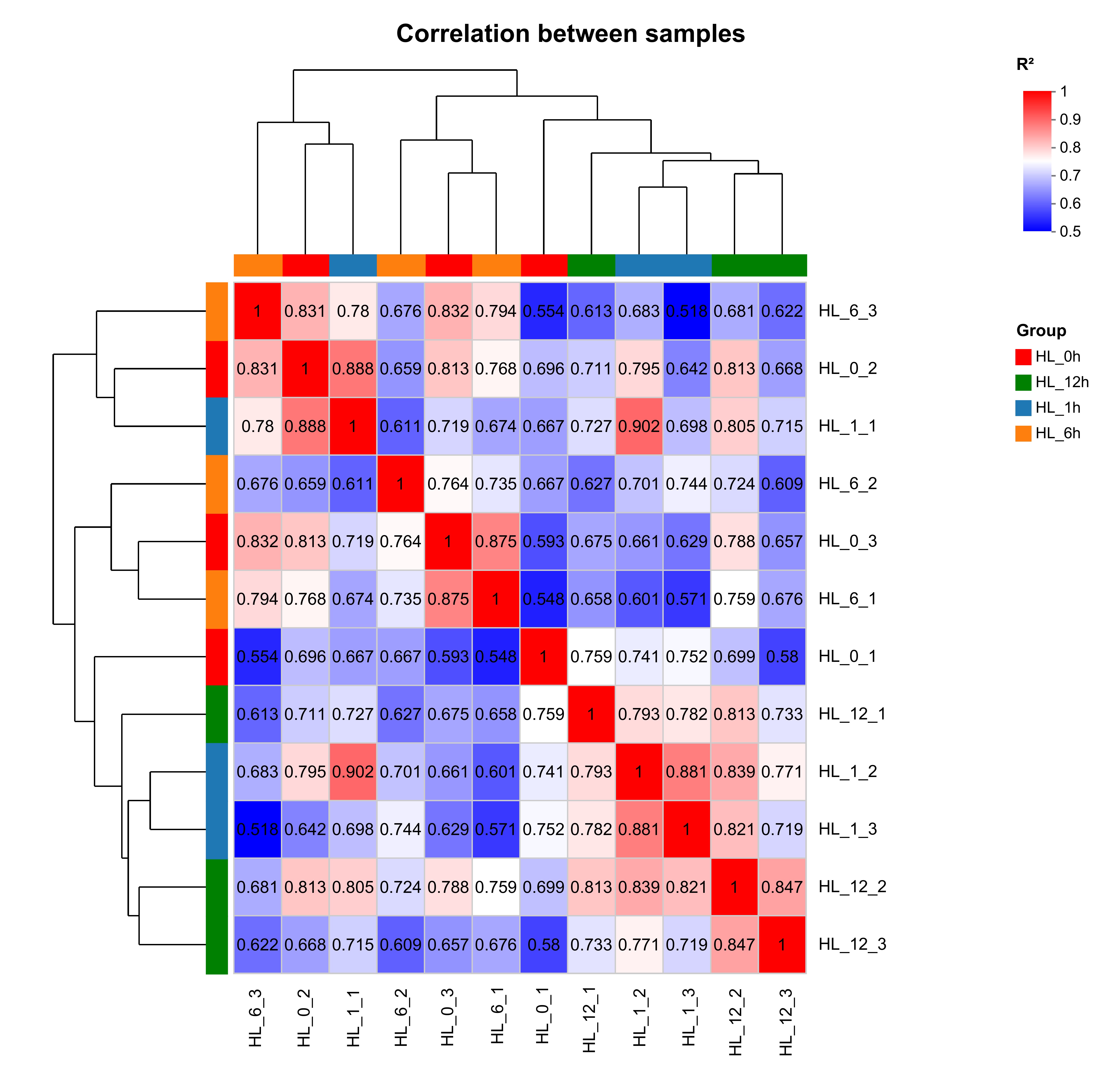

Supplement: Supplementary file 4 — Additional file 4: Fig. S2. Correlation analyses of HL. [file 12870_2025_6599_MOESM4_ESM.jpg]
